# Experimental evaluation of biological regeneration of arable soil: The effects of grass-clover leys and arbuscular mycorrhizal inoculants on wheat growth, yield, and shoot pathology

**DOI:** 10.3389/fpls.2022.955985

**Published:** 2022-08-24

**Authors:** Nichola Austen, Stefanie Tille, Despina Berdeni, Leslie G. Firbank, Martin Lappage, Michaela Nelson, Thorunn Helgason, Ewan Marshall-Harries, H. Bleddyn Hughes, Richard Summers, Duncan D. Cameron, Jonathan R. Leake

**Affiliations:** ^1^Plants, Photosynthesis and Soil, School of Biosciences, University of Sheffield, Sheffield, United Kingdom; ^2^School of Biology, University of Leeds, Leeds, United Kingdom; ^3^Department of Biology, University of York, York, United Kingdom; ^4^RAGT Seeds Ltd., Saffron Walden, United Kingdom; ^5^The Institute for Sustainable Food at the University of Sheffield, Sheffield, United Kingdom

**Keywords:** regenerative agriculture, arbuscular mycorrhizal fungi (AMF), conservation tillage, no-tillage, grass-clover leys, systemic induced resistance

## Abstract

Wheat yields have plateaued in the UK over the last 25 years, during which time most arable land has been annually cropped continuously with short rotations dominated by cereals. Arable intensification has depleted soil organic matter and biology, including mycorrhizas, which are affected by tillage, herbicides, and crop genotype. Here, we test whether winter wheat yields, mycorrhization, and shoot health can be improved simply by adopting less intensive tillage and adding commercial mycorrhizal inoculum to long-term arable fields, or if 3-year grass-clover leys followed direct drilling is more effective for biological regeneration of soil with reduced N fertiliser. We report a trial of mycorrhization, ear pathology, and yield performance of the parents and four double haploid lines from the Avalon x Cadenza winter wheat population in a long-term arable field that is divided into replicated treatment plots. These plots comprised wheat lines grown using ploughing or disc cultivation for 3 years, half of which received annual additions of commercial arbuscular mycorrhizal (AM) inoculum, compared to 3-year mown grass-clover ley plots treated with glyphosate and direct-drilled. All plots annually received 35 kg of N ha^−1^ fertiliser without fungicides. The wheat lines did not differ in mycorrhization, which averaged only 34% and 40% of root length colonised (RLC) in the ploughed and disc-cultivated plots, respectively, and decreased with inoculation. In the ley, RLC increased to 52%. Two wheat lines were very susceptible to a sooty ear mould, which was lowest in the ley, and highest with disc cultivation. AM inoculation reduced ear infections by >50% in the susceptible lines. In the ley, yields ranged from 7.2 to 8.3 t ha^−1^, achieving 92 to 106% of UK average wheat yield in 2018 (7.8 t ha^−1^) but using only 25% of average N fertiliser. Yields with ploughing and disc cultivation averaged only 3.9 and 3.4 t ha^−1^, respectively, with AM inoculum reducing yields from 4.3 to 3.5 t ha^−1^ in ploughed plots, with no effect of disc cultivation. The findings reveal multiple benefits of reintegrating legume-rich leys into arable rotations as part of a strategy to regenerate soil quality and wheat crop health, reduce dependence on nitrogen fertilisers, enhance mycorrhization, and achieve good yields.

## Introduction

Wheat is one of the most important crops globally, serving as a staple cereal for about a third of the world's population (IDRC, 2010). It is the most widely grown arable crop in the UK, covering 38% of the cropped area in 2018 (Defra, 2020). Intensification of wheat production from 1976 to 1996, in Europe and North America, substantially increased yields (Khoury et al., 2014; Pretty and Bharucha, 2014; Spangler et al., 2020; Raven and Wagner, 2021). For example, doubling from 4 to 8 t ha^−1^ in the UK (Defra, 2012), in association with increased chemical inputs from fertilisers, herbicides, fungicides, and growth regulators applied to elite varieties bred for high productivity and disease resistance (Lammerts van Bueren et al., 2011; Hawkesford, 2014; Zetzsche et al., 2020). These “conventional” agricultural practises drove landscape-scale intensification with continuous annual cropping, which replaced mixed farming with rotations including 2- to 3-year legume-rich leys that were either grazed or mown. Previously, these were fed to livestock, and their manure returned to fields, building soil organic matter and fertility between arable crops (Knox et al., 2011). The use of leys in arable rotations, once a common practise in the UK up until the 1960–1970's (Johnston and Poulton, 2018), declined as mixed farming became increasingly uneconomic (Defra, 2020).

For the past few decades, intensive conventional arable production has focussed on short rotations with a small number of the most profitable crops. Winter wheat, barley, and oilseed rape together accounted for 77% of the UK's cropped area in 2018 (Defra, 2020; Harkness et al., 2020). However, since 1996 (Knight et al., 2012; Slater et al., 2021), average yields have plateaued despite a fairly recent world record of wheat yields being achieved in the UK; for example, up to 16.5 t ha^−1^ from one field in 2015 (Hennessy, 2016). The lack of significant yield gain over 25 years has coincided with a period of revolutionary technological advances, including in wheat genetic mapping and marker-assisted breeding to increase yields, and select disease resistance and quality traits (Kuchel et al., 2007; Dhariwal and Randhawa, 2022; Pandurangan et al., 2022). Although some modern varieties have yield potentials approaching 20 t ha^−1^ under optimum conditions, most UK farmers, despite achieving amongst the highest yields in the world, are typically achieving <50% of these potentials. Wheat yields dipped below 7 t ha^−1^ in 2012, for the first time in 20 years, and only reached 7 t ha^−1^ in 2020, which is lower than the 5-year average of 8.4 t ha^−1^ (Defra, 2020).

There is increasing evidence that high-yielding varieties are not achieving their potential due to negative feedbacks caused by intensification. These include the evolution of herbicide-resistant weeds, like blackgrass (Hicks et al., 2018), and repeated breakdown of resistance to fungal pathogens, which are more prevalent in highly nitrogen-fertilised wheat (Zetzsche et al., 2020). Declining organic matter in arable soils (Kirk and Bellamy, 2010) reduces their capacity to store water and nutrients and increases crop vulnerability to droughts and floods. It has been estimated that 80% of agricultural land is experiencing increasingly severe degradation (Pimentel and Burgess, 2013), especially in intensively cultivated soils, which suffer unsustainable erosion losses (Borrelli et al., 2017; Evans et al., 2020). These soil and biological effects on crops are compounded by an increasing frequency of excessive rainfall and drought events, which are linked to climate change (Lowe, 2018; Slater et al., 2021). Extreme weather challenges to arable farming are expected to intensify, with further potential yield declines of 20% due to water stress being predicted (Putelat et al., 2021). In addition, rising costs of chemicals and fertiliser (Baffes and Koh, 2021) due to the escalation of prices of natural gas used for ammonia production, and the depletion of global high-grade rock phosphorus reserves (Alewell et al., 2020), puts increasing pressure on farmers' tight economic margins (FAO, 2022). Furthermore, diminishing returns from oilseed rape break crops (Sieling and Christen, 2015), following the ban of neonicotinoid insecticides and a rise in pyrethroid-resistant flea beetles (Dewar, 2017), have made the widely deployed high-input, short-rotation wheat-rape production system increasingly uneconomic (Agrii, 2016).

The combined environmental and economic pressures on conventional intensive wheat-cropping are motivating an increasing number of farmers to seek more sustainable lower-input crops/cultivars that are potentially more resilient to environmental stress, trading yield for additional beneficial agronomic traits in wheat, and adopting less input-intensive production systems more generally. These include adopting minimal tillage or no-tillage, which reduces fuel use (Holland, 2004), diversifying cropping, and using fewer chemical inputs to reduce costs and improve soil health and biodiversity, thereby harnessing the latent potentials of beneficial soil organisms (Schreefel et al., 2020). There is increasing evidence that high-input arable cultivation and cropping reduces the populations and activities of “ecosystem engineer” organisms that maintain soil structure and functions, including root-symbiotic arbuscular mycorrhizal fungi (AMF) (Helgason et al., 1998; Garcia et al., 2007; Wilson et al., 2009; Cameron, 2010; Oehl et al., 2010) and earthworms (Holden et al., 2019). Both these groups of organisms create water-stable soil aggregates that are important for soil organic carbon sequestration and associated macropores that improve hydrological functioning by faster infiltration and greater water storage capacity, enhancing drought and flood resilience (Wilson et al., 2009; Hallam and Hodgson, 2020; Hallam et al., 2020). Declines in these beneficial soil organisms leave crops increasingly susceptible to competition from weeds (Rinaudo et al., 2010; Veiga et al., 2011) and from attacks by pests and diseases, many of which can cause serious outbreaks in large-scale monocultures grown with abundant fertiliser (Zetzsche et al., 2020). Reduced water-holding capacity and infiltration rates increase both flooding and drought, and these stresses further impact yields by increasing the incidence and severity of diseases. These include leaf blotch *Zymoseptoria tritici*, the most serious disease in the UK, causing yield losses of up to 50% in susceptible wheat varieties (Home-Grown Cereals Authority, 2006), and take-all (*Gaeumannomyces graminis* var. *tritici*), which affects half of the UK wheat crop, with yield losses of 5–20% valued at up to £60 M per year (Home-Grown Cereals Authority, 2006).

Importantly, crop interactions with AMF can activate systemic defence reactions in plants (Khaosaad et al., 2007; Jung et al., 2012; Cameron et al., 2013; Pérez-de-Luque et al., 2017). AMF can help to repel both root pathogens, including take-all (Khaosaad et al., 2007) and shoot pathogens, including powdery mildew (Mustafa et al., 2017). The AM-induced resistance to take-all varies significantly between different varieties of cereal crops (Castellanos-Morales et al., 2011), revealing a trait that may not be selected in conventional crop breeding trials.

The biodiversity and activity of AMF are widely diminished by plough-based crop production systems (Helgason et al., 1998; Oehl et al., 2010; Brito et al., 2012) with even a single ploughing event causing major loss of mycorrhiza (Garcia et al., 2007). This raises the question as to whether wheat can be grown more sustainably by reducing soil disturbance by ceasing inversion ploughing, adopting shallow non-inversion disc cultivation, and regenerating mycorrhizas by adding commercial AMF inoculum (Fernández et al., 2017; Basiru et al., 2021). In addition to direct crop benefits, adding AMF inoculum with compost to wheat crops in Turkey improved soil aggregation and soil carbon sequestration (Ortas et al., 2013). From 1975 to 2013, there are more than 20 reports of AMF inoculum additions to fields in the US, Canada, India, Iran, and China, increasing yields by an average of 20% (Pellegrino et al., 2015). However, these countries have production systems of much lower inputs than the UK, achieving mean yields in 2018 of only 3.1–3.4 tonnes ha^−1^ rising to 5.5 tonnes ha^−1^ in China (Ramadas et al., 2019). To date, there appear to have been no comparable published field trials of AMF inoculum additions to wheat in the UK where chemical inputs and yields are amongst the highest in the world, so the effects of inoculum on wheat yield and crop health on soils that have experienced these intensive production systems remain untested, or unreported.

The extent of soil organic matter depletion and soil degradation in intensively managed agricultural landscapes has been estimated to cost the UK economy over £1.2 b per year (Graves et al., 2015). Therefore, simply reducing tillage intensity and adding microbial inoculants may be insufficient to deliver substantial improvements in soil quality and crop performance. Indeed, the extent of the challenges facing arable farmers has encouraged a rapid rise in the adoption of more radical changes in farming philosophy and practise including agro-ecology and regenerative agriculture practises as seen in the US (LaCanne and Lundgren, 2018) and beyond (Newton et al., 2020; Schreefel et al., 2020). Regenerative agriculture is based on five core principles focused on enhancing and maintaining soil biology, including protecting soil from erosion and surface damage by maintaining the cover of vegetation and crop residues, minimising tillage and soil disturbance, increasing crop diversity, maintaining living root systems all year, and integrating livestock (Newton et al., 2020; Schreefel et al., 2020). Reintegration of legume-rich leys into arable rotations meets all five principles of regenerative agriculture when grazed, or mown and fed to livestock with manure returns. However, even without livestock integration or silage off-take and manure returns, recent studies of mown grass-clover leys reintroduced into arable rotations have shown rapid regeneration of several key indicators of soil health. These include an increase in earthworm populations (Prendergast-Miller et al., 2021), improvements in soil structure, soil aggregation, water-holding capacity, and infiltration rates over 1–2 years (Puerta et al., 2018; Hallam et al., 2020; Berdeni et al., 2021). These soil quality improvements increased wheat crop resilience to flooding and moderate drought in an outdoor mesocosm study (Berdeni et al., 2021), but the effects on mycorrhiza and crop pathology were not investigated. A number of recent studies have begun to investigate the effects of establishing leys for soil quality regeneration (Albizua et al., 2015; Berge et al., 2016; Zani et al., 2020; Berdeni et al., 2021; Prendergast-Miller et al., 2021). However, the extent to which leys regenerate soil biology, fertility, and crop yields have received little attention in modern intensive arable farming systems. Older studies predating intensification (e.g., Hanley et al., 1964) may no longer be relevant given the unprecedented subsequent changes. These include depletion of soil organic matter (Graves et al., 2015), accumulation of P fertiliser in soils over the past 50 years (Withers et al., 2001), and use of modern fertilisers, herbicides, fungicides, and growth regulators on newer and higher yielding varieties of wheat.

Legume-rich leys, typically containing clovers and grasses, and legume crops, such as Fava beans (*Vicia faba*), are important sources of biologically fixed nitrogen, which build soil fertility in organic farming systems and can substantially reduce nitrogen fertiliser requirements in conventional farming (McKenna et al., 2018). In the UK, synthetic nitrogen fertiliser contributes 46% of the environmental footprint of a loaf of bread (Goucher et al., 2017), but typically less than 50% of the N fertiliser applied to wheat at standard application rates is taken up by the crop, (Hawkesford and Riche, 2020). Nitrogen-use efficiency varies by genotype and decreases below 40% with N-addition rates above 200 kg ha^−1^ (Hawkesford and Riche, 2020). The excess not taken up by the crop is mainly lost to water and air pollution, to which farming makes major contributions (Defra, 2019). The reintegration of legume-rich leys into arable rotations would be expected to reduce fertiliser use and associated costs and pollution. The legume species may also selectively promote the growth and activity of a subset of AMF that may form symbioses with a following wheat crop, providing the soil is not disturbed as has been seen in Portugal (Brígido et al., 2017). However, the traditional method of reverting from ley to arable cultivation has been to plough and harrow to destroy the existing vegetation that could compete with the crop, deplete potential invertebrate herbivores, such as slugs and leatherjackets, and to mineralize accumulated organic nitrogen. This process breaks down soil aggregates and oxidises much of the accumulated organic matter, adversely impacting the beneficial soil organisms, and thereby reverses the improvements in soil health achieved by the leys, requiring leys to be reinstated after a few years (Knox et al., 2011). To better preserve gains in soil quality and soil organisms, it may be possible to revert from ley to arable cropping using broad-spectrum herbicide followed by direct drilling, but there are some reports that the most widely used herbicide, glyphosate, can be detrimental to mycorrhiza inoculum potential (Druille et al., 2013a,b).

Following the long history of highly selective breeding of wheat, there has been evidence of genotypic variation in hosting AMF (Hetrick et al., 1992, 1993, 1995; Lehnert et al., 2017, 2018) and in resistance to pathogens (Laidig et al., 2021). To evaluate field management practises on AM colonisation in wheat roots and potential pathogen resistance, trials ideally need to include a contrasting range of genotypes to encompass this variation (Hohmann and Messmer, 2017). In the present study, we selected four double-haploid offspring lines from the UK winter wheat reference population, Avalon x Cadenza, that are genetically well-characterised and have been used for the development of quantitative trait loci and genetic mapping studies (Ma et al., 2015). In an unpublished preliminary multi-site field study conducted in Eastern England by RAGT Seeds, there appeared to be marked differences in mycorrhiza competence between lines in this mapping population; from these, we selected three pairs of putatively “high” and “low” mycorrhizal competence lines for our study.

The overarching aim of this study was to investigate enhancing winter wheat yields, mycorrhizal colonisation and disease resistance under reduced fertiliser applications on typical lowland arable land in eastern England that has been intensively cultivated and annually cropped for decades. Two approaches to mycorrhizal enhancement under reduced nitrogen and no phosphorus fertiliser inputs were investigated and compared. Firstly, the effects of adding commercial mixed species of mycorrhizal inoculum to six wheat lines, sown in plots that were ploughed or disc-cultivated, were compared to uninoculated control plots under the two tillage intensities over 3 years in succession. Here, we present data for the final year. Secondly, plots of 3-year mown grass-clover ley established in the same field were direct drilled with the same six wheat lines after herbicide treatment in the same final year of the study. This multi-factorial experiment determines the extent to which mycorrhization and disease incidence changes with the implementation of this regenerative agriculture approach to enhance soil biology and functioning.

These studies were designed to test the following hypotheses:

1. Adding a commercial mixed species UK-sourced mycorrhiza inoculum to soil that is likely to be mycorrhiza-depleted after decades of conventional intensive annual cultivation cropping can increase mycorrhization and yield and reduce shoot disease incidence in wheat.
2. The benefits of mycorrhizal inoculum addition to wheat will be greater in ploughed and harrowed plots than in disc-cultivated plots, as the native mycorrhizal inoculum will be most depleted by the most intensive cultivation.
3. Mycorrhization will be substantially increased, yields improved, and fertiliser requirements reduced in wheat lines direct drilled into 3-year grass-clover leys deployed to regenerate soil biology and build fertility, but shoot pathogens may be more prevalent due to likely transfer from the ley species.
4. Wheat genotype has a significant effect on wheat mycorrhizal colonisation responses to inoculation, tillage methods, or direct drilling into ley, as well as on shoot pathology and yields, giving genotype-specific outcomes to hypotheses 1–3 above.

Our research is highly timely and strategically important due to escalating fertiliser costs and the rapidly growing interest of farmers and policymakers in more sustainable farming practises, such as regenerative agriculture. This is reflected in the new Environmental Land Management farm payments system in the UK, aiming to provide subsidies to farmers on the basis of public money for public goods (Defra, 2021a), and moves to revise the common agricultural policy in the EU. Critically, both policymakers and practitioners urgently need better evidence of how to regenerate soil health and improve crop production to guide management decisions.

## Materials and methods

### Field experiment set up

**Figure 1:**
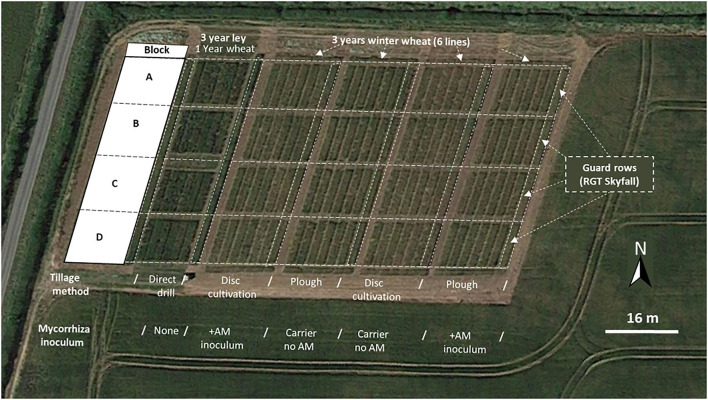
Aerial view (June 2018) of the experimental plots arranged in four blocks (A-D) running downslope at Spen Farm, Tadcaster (53° 51' 44” N; 1° 20' 35”W). The image shows the wheat direct drilled into a 3-year ley compared to plots that have been cultivated and cropped with wheat for 3 successive years, with Skyfall wheat guard rows and the surrounding field which is not part of the trial. Map data © 2018 Google.

The field trial was conducted at The University of Leeds farm, Tadcaster. Spen Farm is a commercial, conventionally managed mixed arable and pasture farm. The main soil type is a well-drained, loamy, calcareous brown earth of the Aberford series in the Calcaric Endoleptic Cambisols (Cranfield University, 2019), which is underlain by dolomitic limestone of the Cadeby formation (Smith et al., 1986). This is an important soil type on gently sloping Permian and Jurassic limestone, covering 1,125 km^2^ of mainly arable land in lowland areas of England and Wales. The field used for the wheat trial experiments is found at coordinates 53° 51' 44” N; 1° 20' 35”W within the farm (see Figure 1 for an aerial view of the field in relation to the farm).

To test the effects of both mycorrhizal association and tillage method on winter wheat (*Triticum aestivum*), the plants were planted in a grid system. Supplementary Figure S1 is a schematic of the experimental set up, which was repeated for three successive years from autumn 2015, with the same treatments applied to each plot. In the main section of the field, tillage methods were separated into two treatments: disc cultivation (minimal tillage) and conventionally ploughed soil. Both tillage treatments had either a commercial arbuscular mycorrhiza fungal inoculum (Mycorrhiza Rootgrow™ Professional, PlantWorks, UK) added to them or the carrier substrate without the AM inoculant (PlantWorks, UK). The inert clay carrier acts as a substrate for the fungi to grow through and contains bio-additives which enhance mycorrhizal colonisation, such as chitin (from crustaceans), alginates (from seaweed), and humates (from decomposed organic matter). Each tillage/AM treatment block was separated by the addition of guard rows of winter wheat (RGT Skyfall, RAGT seeds, Essex, UK). Each treatment block was sown with two parental and four double haploid wheat lines (Avalon, Cadenza, AxC 22, AxC 53, AxC 57, and AxC 69). Each of the wheat genotypes were randomised in each treatment and split into four blocks running downslope (each 9 m x 1.5 m, 16 blocks in total) for replication. The winter wheat genotypes were sown annually between October-November in the same plots from 2015 to 2017.

### Cultivation and sowing strategies

#### Disc cultivation minimal tillage plots

The disc-cultivated plots (see Supplementary Figure S1 for details) were cultivated using the cultivators employed by contract farmers managing the adjoining arable fields, and varied from year to year, with a Lemken Heliodor in 2015, a Sumo Trio in 2016, and a Väderstad Topdown in 2017, the latter being the year for which we report the results here.

#### Ploughed plots

The trial plots that were mouldboard-ploughed were cultivated with a five-furrow reversible plough in 2015 and a Dowdswell DP 8A 2 furrow reversible plough in 2016 and 2017. After ploughing and in preparation for sowing, the ploughed areas were power-harrowed followed by a pressing roller (Howard Roterra HK20) (see Supplementary Figure S1 for details of plots).

#### Seed-drilling and quantities

In 2015, the seeds of the different wheat lines for sowing in plots were provided from plots sown, harvested, and prepared by RAGT seeds (Saffron Waldon, UK). For both the minimally tilled plots and the ploughed plots, the wheat seeds were sown with a Wintersteiger Øyjord plot drill. Seeds for each plot (9 × 1.5 m subplot) were weighed to equate to 400 seeds m^−2^ (4,800 seeds per subplot). In 2017, saved seeds from the first-year harvest were used, except AxC 53, which had a poor germination rate and was thus supplemented with saved seeds from the second year's harvest. The seed quantities in 2017 were adjusted to consider the percentage germination rate for the seeds of each wheat genotype. See Supplementary Table S2 for details of seed weights for each subplot.

#### Mycorrhiza inoculum application

The mycorrhizal inoculum was RootGrow Professional, supplied by Plant Works UK, and was specified as a five-species mix comprising *Funneliformis mosseae, F. geosporus, Claroideglomus claroideum, Rhizophagus irregularis*, and *Glomus microagregatum*, with a total propagule number of 1.6 million per litre, supplied in a granular form with zeolite particles. The inoculum was grown for each of the 3 years from 2015 to 2017 so that fresh inoculum was applied after each annual sowing. The inoculum was added at the same time as the seeds were drilled using a Stocks Micro Meter, mounted onto the front of the Øyjord Plot drill. The uninoculated control plots received the sterile zeolite carrier at the same rates as inoculum, which was 204 g m^−2^ in year one, 99 g m ^−2^ in year two, and 204 g m^−2^ in the final year (2017–18; Supplementary Table S2).

### Wheat genotype selection

The parental winter wheat lines, Avalon and Cadenza, are dwarf and non-dwarf varieties, respectively. From these two parent lines, four Avalon x Cadenza (A x C) double haploid lines were chosen from the UK reference population [Wheat Genetic Improvement Network (WGIN)]. The mapping population was previously sequenced by SNPs using the Affymetrix (Thermo Fisher, UK) Axiom^®^ wheat breeders genotyping array (Bai et al., 2013; Allen et al., 2017). Prior to the study that we report here, a trial study was undertaken through a Technology Strategy Board-funded grant in collaboration with RAGT Seeds that provided an initial pot experiment to investigate the effects of conventional vs. organic management, mycorrhiza, and wheat genotype on wheat yield. Following this, a field trial was conducted in Cambridgeshire, in which 50 A × C wheat lines were sown into conventional and organic arable fields, and their roots were sampled during the initiation of flowering, and mycorrhization was estimated using the unpublished biomarkers established from the pot experiment.

### Establishment of 3-year ley and subsequent wheat drilling

A grass-clover ley was established in parallel, alongside the main experimental plot, 20 m away from the field margin (Figure 1), running down the slope of the field (see Table 1 and Supplementary Figure S1 for experimental set-up). In November 2014, ryegrass (*Lolium perenne*, and *Lolium x boucheanum*) was sown. In March 2015, the grass was mown short, and clippings were removed and then oversown with white and red clover (*Trifolium repens* and *Trifolium pratense*). The grass-clover ley was maintained by mowing 4–5 times per year and removal of clippings, until being sprayed off with glyphosate in late October 2017.

**Table 1:** Parameters and time periods for treatment application for the four-year field trial of winter wheat.

		**Ley (direct drilled)**	**Disc cultivated (+) AMF**	**Ploughed (-) AMF**	**Disc cultivated** **(-) AMF**	**Ploughed (+) AMF**
Years (with wheat crop)		2017–2018	2015–2018	2015–2018	2015–2018	2015–2018
Mycorrhiza Inoculum		No	Yes	No	No	Yes
Wheat Lines		Avalon, Cadenza, AxC22, AxC53, AxC57, AxC69	Avalon, Cadenza, AxC22, AxC53, AxC57, AxC69	Avalon, Cadenza, AxC22, AxC53, AxC57, AxC69	Avalon, Cadenza, AxC22, AxC53, AxC57, AxC69	Avalon, Cadenza, AxC22, AxC53, AxC57, AxC69
Fertiliser	2016	None	YaraBela™	YaraBela™	YaraBela™	YaraBela™
(35 kg N ha ^−1^)	2017	None	YaraBela™ Nitram^®^	YaraBela™ Nitram^®^	YaraBela™ Nitram^®^	YaraBela™ Nitram^®^
	2018	Nitram^®^	Nitram^®^	Nitram^®^	Nitram^®^	Nitram^®^

Table details the wheat lines established between 2015–2018, the cultivation type, whether there was an AM inoculum or sterile carrier added, and the fertiliser application rates.

A month later, the ley was direct-drilled (using a plot drill based on a Sim-tech Aitchison grassland regeneration drill operated by Envirofield Ltd), with the winter wheat lines arranged in parallel blocks to those in the adjacent experiment and sown at a matched seed density at the same time as the main plots were sown with wheat for the third and final year (2017–18).

### Application of fertilisers and pest treatments

#### Fertilisers

Fertiliser was applied to all the plots in the main field experiment using a hand-held spreader. Both YaraBela™ (34.5% N, 17.3% nitric, 17.2% ammoniacal) ammonium nitrate fertiliser (Yara, Grimsby, UK) and Nitram^®^ (34.5% ammonium nitrate) ammonium nitrate fertiliser (CF Fertilisers, Cheshire, UK) were applied during the 3-year field plot experiment at an annual rate of 35 Kg N ha^−1^ (Table 1).

#### Herbicides

The fallow rows between the replicate blocks (running north to south), the fallow row between the main field plots, and the ley strip were treated with glyphosate (Glyphos^®^ Headland Agrochemicals Ltd, Flintshire, UK) and sprayed at intervals using a knapsack sprayer (3 L/ha in 200 L). The mix contained 360 g/L glyphosate (present as 41.2% (w/w) of the isopropylamine salt). The wheat plots were treated annually with several different herbicides (see Supplementary Table S3 for a list of herbicides applied each year).

#### Fungicides

No fungicides were applied during the field trials as this might have an impact on both the commercial inoculum that was added to the disc cultivated and ploughed plots and the pre-existing/newly established fungal species that may have been present in the ley and arable soils.

#### Molluscicides and insecticides

Ferric phosphate Sluxx HP (Certis, Cambridgeshire, UK) pellets were applied using a handheld spreader for each of the 3 years in the main field plots to provide protection during wheat-seedling establishment (Supplementary Table S4). Insecticides were not used during the experiment as they were not deemed necessary.

### Sampling strategies

#### Sampling for AMF colonisation of roots

The colonisation of wheat roots by arbuscular mycorrhizal fungi was assessed for roots in soil cores collected in June from both the main field plot sites and the direct-drilled ley plots. Three cores were taken from each plot (top, middle, and bottom) at depths of 0–10 cm and 10–20 cm (corer diameter 4.5 cm and length 20.5 cm). The roots were carefully removed from the soil on a sieve and washed with water, and the roots pooled from the two different depths were weighed and subsequently frozen at −20°C, ready for analysis.

#### AMF-scoring of roots

Quantification of AM colonisation was conducted on the frozen root samples after thawing, with 13 pieces of fine roots selected, cut to 1 cm in length, and stained. The staining method was adapted from Vierheilig et al. (1998) and involved clearing roots in 10% (w/v) KOH (80°C, 35 min), then rinsing with tap water, acidifying with 10% (v/v) HCl (2 mins) and, again, rinsing with water. Cleared roots were stained in 5% (v/v) ink-acetic acid solution (80°C, 30 min), rinsed with tap water, and destained in 5% (v/v) acetic acid (30 min), before a final rinse before mounting in 50% glycerol on glass slides for microscopy. Mycorrhizal colonisation was calculated by percentage root length colonisation based on the magnified intersection method (McGonigle et al., 1990). Counts were made at 100 random locations on the 13 fine root lengths per sample, with the presence/absence of arbuscular fungal hyphae, vesicles, and arbuscules recorded.

### Plant pathology

Sooty ear mould, which is caused by several fungi, typically *Cladosporium* and *Alternaria* spp., was scored immediately before the wheat harvest in 2018, by counting the numbers of blackened ears infected within each plot, with two observers scoring the same plots and their counts being pooled and averaged. Where counts between observers differed by more than 10%, the plots were rescored. The disparities between observers were greatest in the most infested plots. Counts per plot were expressed per unit area, considering the actual area of the crop in each plot. Earlier in the spring the wheat plants were checked and scored for yellow-rust, powdery mildew, and *Zymoseptoria*, following protocols used in the previous 2 years in the same plots, but in 2018 infection levels of all these fungi were very low, and showed no consistent effects of treatments, so the scoring was not completed, and the data are not presented.

### Harvest

The final harvest took place in July 2018 for the main field experiment and the adjacent plots, in which wheat had been direct drilled into the former 3-year ley. Prior to harvest, but once the grain was mature, the height of the wheat in each plot was measured at 10 points and an average was taken. Grain yields from each plot were measured using on-board weighing of a Sampo Rosenlew 2010 plot combine harvester with a moisture meter, and the grain was collected in paper sacks. Straw and chaff weights are not reported.

### Statistical analyses

All statistical analyses were performed using Prism version 9.2.0 (GraphPad Prism version 9.2.0 for Windows, 2021). Differences between tillage method or genotype and soil properties or plant performance were resolved by a one-way ANOVA with Tukey's HSD *post-hoc* tests to calculate significant differences (*P* < *0.05*). Two-way ANOVA was used to analyze data with both tillage type (ley-direct drilled, disc, ploughed) and wheat line (Avalon, Cadenza, AxC 22, AxC 53, AxC 57, AxC 69) as the two factors. Both the independent and interactive effects of tillage type and wheat line were investigated. Tukey's HSD *post-hoc* tests were used to determine significant differences between the treatments (*P* < *0.05*). To undertake full factorial analysis of the permanent arable plots, testing for effects of wheat genotype, presence or absence of commercial mycorrhizal inoculum, and ploughing vs. disc cultivation, and their interactions, we conducted 3-way ANOVA, excluding the ley data which was not part of that factorial design. To investigate the consistency of the treatment effects on yields reported for 2018, concerning the two previous years of wheat growing on the permanent arable plots (i.e., excluding the ley plots) we used a three-way ANOVA. This tested the effect on grain yield of mycorrhizal inoculum, ploughing vs. disc cultivation, and year of harvest (2016–18), and the interactions between these factors, and is presented in full in the supplementary information (Supplementary Table S2).

## Results

### Wheat performance direct-drilled into ley compared to continuous cropping with ploughing or disc cultivation

**Figure 2:**
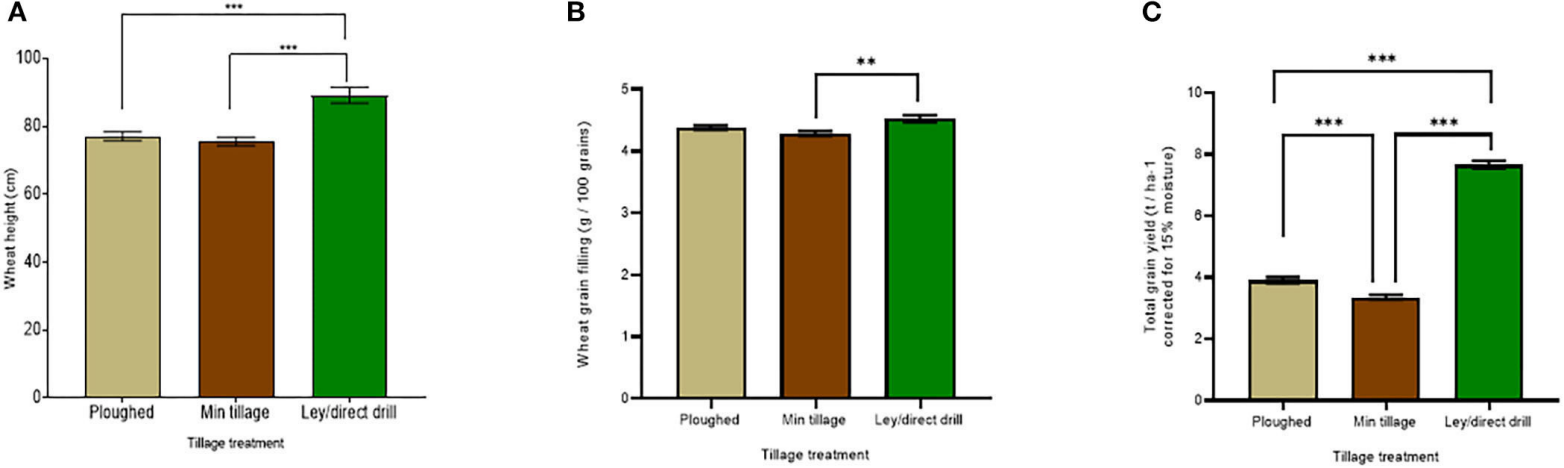
Ley and tillage effects on wheat yields in the 2018 harvest, averaging across all six genotypes and across the treatments with and without mycorrhiza inoculum added. Bars represent the tillage treatments: ploughed, minimally tilled, and direct drilled into ley. **(A)** wheat tiller height (cm) (ploughed *n* = *48*, min tillage *n* = *52*, and ley *n* = *24*), **(B)** grain-filling (g / 100 grains) (ploughed *n* = *48*, min tillage *n* = *48*, ley *n* = *24*), and **(C)** total grain yield (corrected for 15% moisture, t / ha ^−1^) (ploughed *n* = *48*, min tillage *n* = *47*, and ley *n* = *24*). The error bars show ± 1 SE. Results of Tukey's HSD test results of significant differences are shown (*p = *0.05;* **p = *0.001;* ***p < *0.0001)*.

Winter wheat yield in 2018 was highly significantly improved (*p* < *0.001*) when grown by direct drilling into the ley compared to the disc and ploughed plots, in which wheat had been grown for 3 years in succession (Figure 2C). Across all wheat lines, the mean yields for the ley, ploughed, and minimally tilled plots were 7.7, 3.9, and 3.4 t ha ^−1^, respectively. Tukey's *post-hoc* HSD tests (see Supplementary Table S1 for statistical results) showed significant differences (*p* < *0.001*) between the ley and disc cultivated, the ley and the ploughed, and the minimally tilled and the ploughed soils. The yield improvements in the former ley were also reflected in the significantly taller plants (*p* < *0.001*) compared to the ploughed and minimally tilled plots (means = 89.2, 77.1, and 75.6 cm, respectively; Figure 2A). Although the wheat grown with ploughing was slightly taller than under disc cultivation, this effect was not significantly different (see Supplementary Table S1 for full statistical results). Like grain yield, grain filling (weight of 100 seeds) was the highest in the ley (4.5 g per 100 grains), compared to 4.4 g per 100 grains in the ploughed plots, and 4.3 g per 100 grains in the disc-cultivated plots (Figure 2B). However, only the latter was significantly less than for the ley (*p* = *0.0004*) (see Supplementary Table S1 for statistics).

### Mycorrhizal and pathogenic fungi of wheat direct-drilled into 3-year ley compared to continuous tilling and cropping

**Figure 3:**
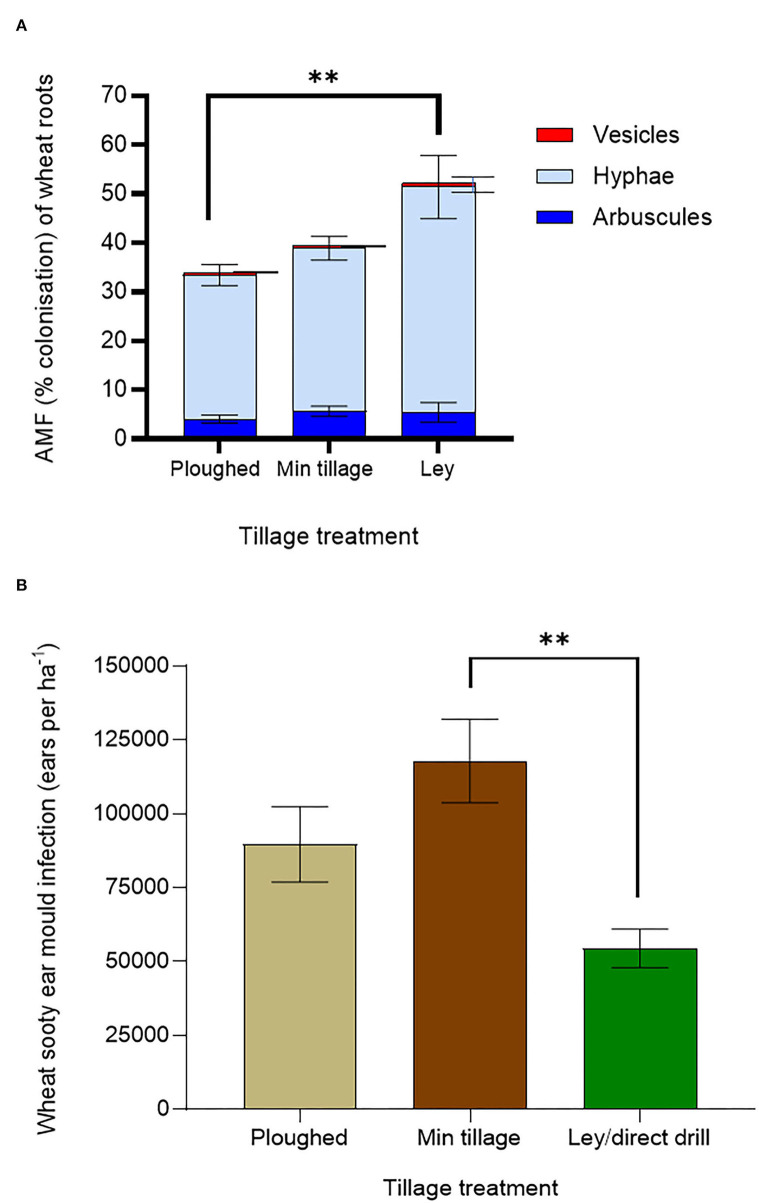
Effects of ploughing, disc- cultivation, and direct drilling into a 3-year ley, on wheat, averaging across all 6 genotypes, and across the treatments with and without mycorrhiza inoculum added, on **(A)** AMF colonisation [expressed as % root length colonisation by arbuscules, hyphae, and vesicles (ploughed *n* = *39*, min tillage *n* = *39*, ley *n* = *5*)] and, **(B)** sooty ear mould (infected ears ha ^−1^) (ploughed *n* = *48*, min tillage *n* = *52*, and ley *n* = *24)*. The error bars show ± 1 SE and are displaced to the right for vesicles for clarity. Results of significant Tukey's HSD test results for differences between means are shown (*p = *0.05;* **p = *0.001;* ***p < *0.0001*).

To understand possible drivers of improved crop performance in the former ley treatments, we assessed mycorrhizal colonisation and shoot pathology (Figures 3A,B). Mycorrhization was highest in the ley (Figure 3A), with a mean of 52.4% for total fine root length colonised, compared to 40.7% colonisation in the minimally tilled, and only 35.8% in the ploughed plots (in both cases averaging across control and AM- inoculated plots); the difference between the ley and ploughed treatments being significant (*p* < *0.05*). The 12.8% decrease in mycorrhizal colonisation from the disc cultivated to ploughed soils was not statistically significant.

Sooty ear mould was most abundant in the disc cultivated and ploughed plots and lowest in the ley (Figure 3B), which was less than half the infection frequency of the disc-cultivated plots (*p* = *0.0108*). Although not significant, the prevalence of the disease in the ploughed plots was 65% higher than in the ley. Disease scores of sooty ear mould in the disc-cultivated plots were 24% higher than in the ploughed plots (Figure 3B).

### Wheat performance with ploughing or disc cultivation and addition of commercial mycorrhizal inoculum, compared to direct drilling into a ley

**Figure 4:**
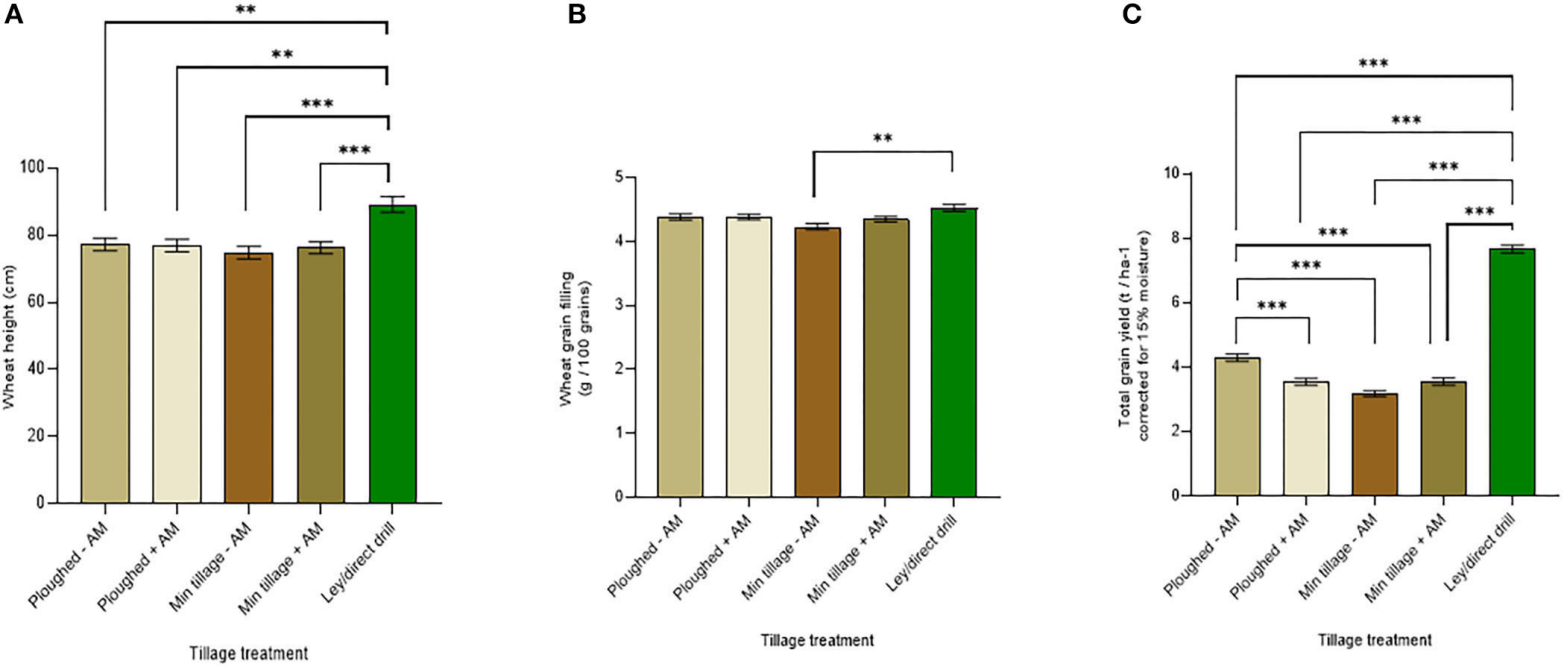
Wheat performance averaged across genotypes on ploughed and minimally tilled plots, with added sterile carrier (- AM) or commercial AMF inoculum (+ AM) compared to direct drilling into ley at the 2018 harvest. **(A)** wheat tiller height (cm) (ploughed – *n* = 24, ploughed + *n* = 24, min tillage – *n* = 24, min tillage + *n* = 28, and ley *n* = 24), **(B)** grain weight (g / 100 grains) (ploughed – *n* = 24, ploughed + *n* = 24, min tillage – *n* = 24, min tillage + *n* = 23, and ley *n* = 24), and **(C)** total grain yield (corrected for 15% moisture, t ha ^−1^) (ploughed – *n* = 24, ploughed + *n* = 24, min tillage – *n* = 24, min tillage + *n* = 24, and ley *n* = 24). The error bars show ± 1 SE. Results of significant Tukey's HSD test results of significant differences are shown on the graphs *p = *0.05;* **p = *0.001;* ***p < *0.0001*).

A major goal of the research was to establish whether adding commercial mycorrhizal inoculum could facilitate increased wheat mycorrhization under continuous cultivation and cropping by ploughing or disc cultivation over several growing seasons (2015–2018) when compared to an herbal ley (see Supplementary Figure S2 for main field grain yields, and Supplementary Table S2 for the associated three-way ANOVA). Mycorrhizal inoculum did not affect the height of the wheat when mature (Figure 4A), while the wheat grown on the ley was significantly higher than the other treatments (*p* < *0.0005*). The greatest difference in plant height was between the wheat grown in the ley and the disc-cultivated soil without inoculum, where the ley increased crop height by 17.6%, averaging across the genotypes.

Similarly, on averaging across genotypes, there was no significant effect of mycorrhizal inoculation on wheat grain filling (Figure 4B). However, the ley showed significantly greater grain filling with 4.5 g per 100 grains compared to the lowest performing treatment (disc cultivation with no mycorrhizal inoculum) with only 4.2 g per 100 grains (*p* = *0.0005*).

Wheat yields averaged across genotypes showed significant differences due to mycorrhiza inoculation, tillage treatments, and, especially the ley, which gave by far the highest yields (Figure 4C). Notably, the highest yields in the continuously cropped plots were seen with ploughing without mycorrhiza inoculation at 4.3 t ha^−1^. Indeed, adding mycorrhizal inoculum to ploughed plots caused a significant reduction (*p* < 0.0001) in yield to 3.5 t ha^−1^. Yields under disc cultivation were consistently lower than with ploughing and tended to be slightly, but not significantly increased (from 3.2 to 3.3 t ha^−1^), by mycorrhizal inoculum. There was no significant difference between the ploughed and disc-cultivated soils with added inoculum, and no significant difference between ploughed plots with mycorrhizal inoculum and disc-cultivated soils with no added mycorrhiza.

#### Tillage and mycorrhiza inoculation effects on mycorrhizal abundance in wheat grown in arable soils in comparison to direct drilling into a grass-clover ley

**Figure 5:**
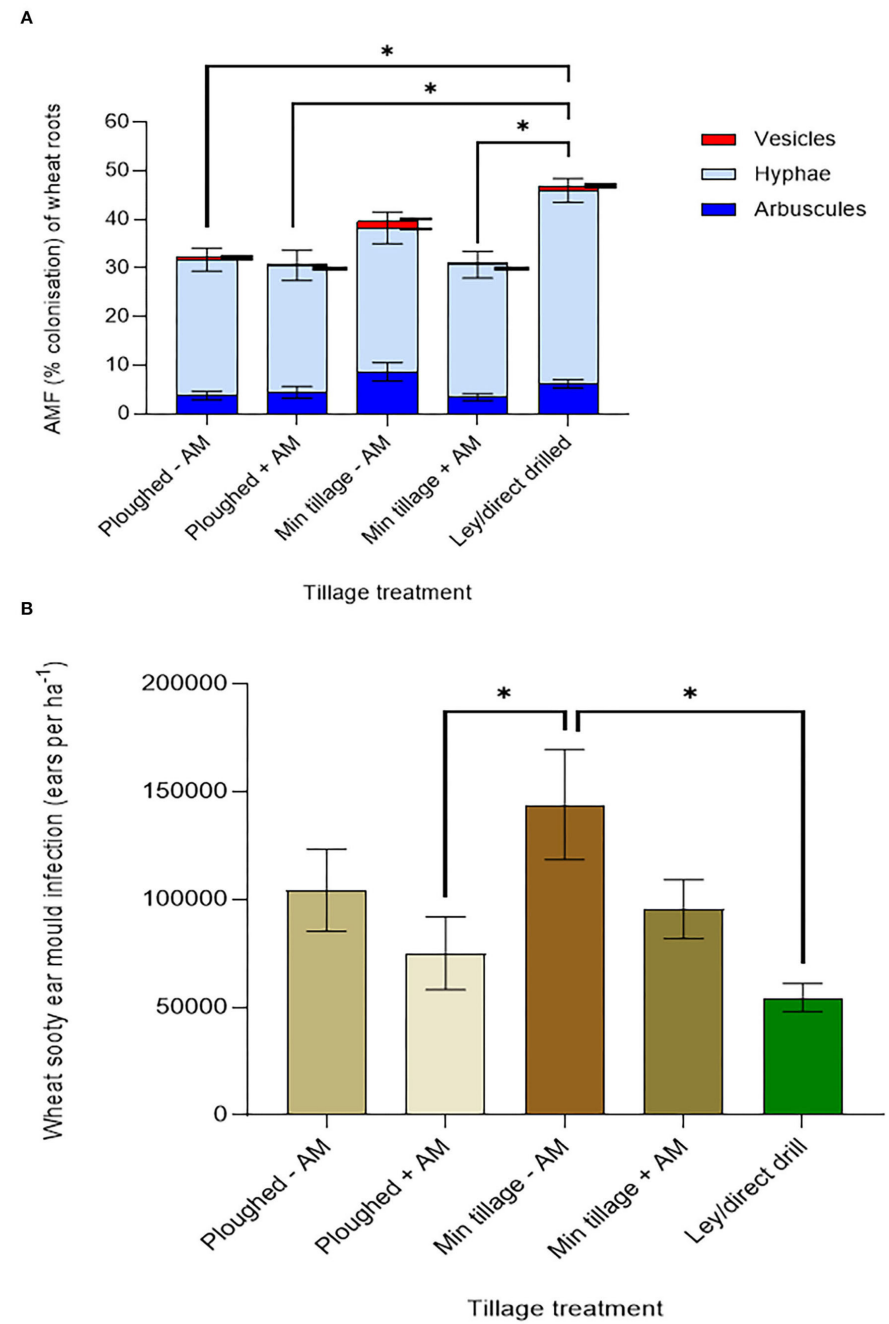
Wheat performance, averaging across genotypes, on ploughed and minimally tilled plots, with added sterile carrier (- AM) or commercial AMF inoculum (+ AM) compared to direct drilling into a 3-year ley at the 2018 harvest. **(A)** Mycorrhizal abundance (% colonisation of wheat roots by hyphae, arbuscules, and vesicles) (ploughed and disc cultivated *n* = 24, ley *n* = 14), and **(B)** Sooty ear mould (infected ears ha ^−1^) (*n* = 24 in all treatments). The error bars show ± 1 SE. Results of significant Tukey's HSD test results of significant differences are shown on the graphs (*p = *0.05;* **p = *0.001;* ***p < *0.0001*).

Averaging across wheat genotypes, the addition of mycorrhizal inoculum to both ploughed, and especially disc-cultivated plots, tended to reduce rather than increase mycorrhization in wheat (Figure 5A). The highest mycorrhization in the continuously cultivated plots was seen in the disc-cultivated plots, to which no live inoculum was added, and it was only this treatment, in which mycorrhization was overall not significantly different from that seen in the ley, which showed the highest proportions of root length colonised, especially by hyphae.

#### Tillage and ley effects on plant health: Results of disease scoring for sooty ear mould (*Cladosporium* or *Alternaria* spp.)

The addition of mycorrhizal inoculum to the continuously cultivated plots that were either ploughed or disc cultivated, in both cases tended to reduce ear mould infections (Figure 5B) suggesting disease suppression by the inoculum. Disc-cultivated plots tended to have higher infection rates than ploughed plots. The highest instances of sooty ear mould were found in wheat grown in the disc cultivated soil with no mycorrhiza inoculum added, while the ley supported the least colonisation, a fall of 90% compared to the highest mean (*p* = *0.0041*).

### Wheat genotypic effects on growth and mycorrhization

**Figure 6:**
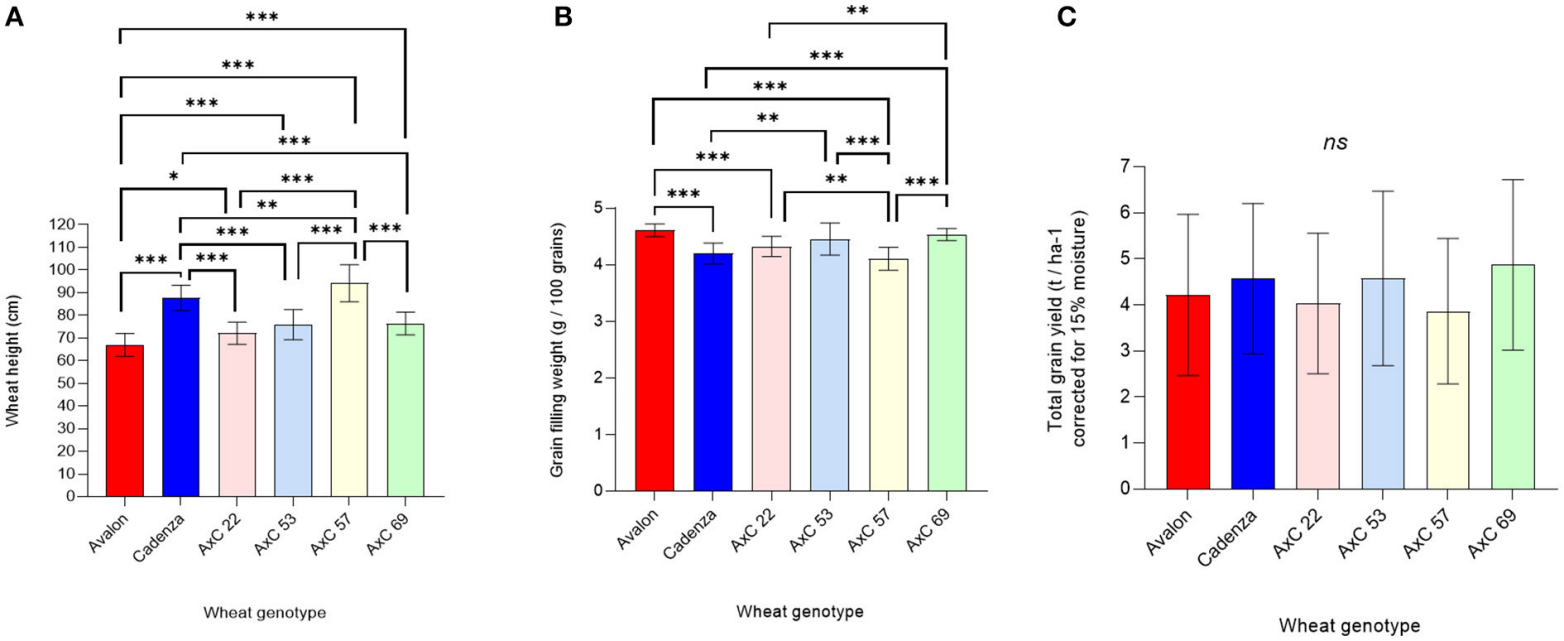
Genotype effects of wheat lines Avalon, Cadenza, AxC22, AxC53, AxC57, and AxC69 on wheat performance, averaging across mycorrhiza, tillage and ley / arable treatments on **(A)** wheat tiller height (cm) (Avalon *n* = 29, Cadenza *n* = 29, AXC22 *n* = 21, AXC53 *n* = 22, AXC57 *n* = 20, and AXC69 *n* = 21), **(B)** grain weight (g / 100 grains) (Avalon *n* = 20, Cadenza *n* = 20, AXC22 *n* = 20, AXC53 *n* = 20, AXC57 *n* = 20, and AXC69 *n* = 20), and **(C)** total grain yield (corrected for 15% moisture, t ha^−1^) (Avalon *n* = 29, Cadenza *n* = 29, AXC22 *n* = 21, AXC53 *n* = 22, AXC57 *n* = 20, and AXC69 *n* = 21). The error bars show ± 1 SE. Results of significant Tukey's HSD test results of significant differences are shown on the graphs (*p = *0.05;* **p = *0.001;* ***p < *0.0001*).

The parental lines, Avalon and Cadenza, have contrasting height phenotypes, as Avalon, which grew to only 67 cm on average, has dwarfing genes (Figure 6A). The progeny lines from the parents also varied in height phenotype, with AxC 57 being the tallest (94 cm), followed by Cadenza (88 cm), and AxC 69, AxC 53, and AxC 22 being intermediate between Cadenza and Avalon in height and significantly different to both parents (Figure 6A).

Grain filling was also affected by genotype, with Avalon producing significantly heavier grains than Cadenza (Figure 6B), while AxC 22, AxC 53, and AxC 69 achieved intermediate grain filling values that were significantly different to both parents in most cases. AxC 57 had the lowest individual grain weights, but these were not significantly different to that of the parent Cadenza. The genotype effects on grain filling were much larger than the effects of tillage or mycorrhizal inoculum (c.f. Figures 4B, 6B).

Averaging across all treatments, grain yields showed no significant genotypic differences (Figure 6C) but high variances; some of which will be due to treatment effects, so these data need to be interpreted with caution. A full expansion of genotype effects by all treatment combinations is given later.

#### Effect of selected wheat genotypes on mycorrhizal competence in arable soils with added commercial inoculum in comparison with a grass/clover ley

**Figure 7:**
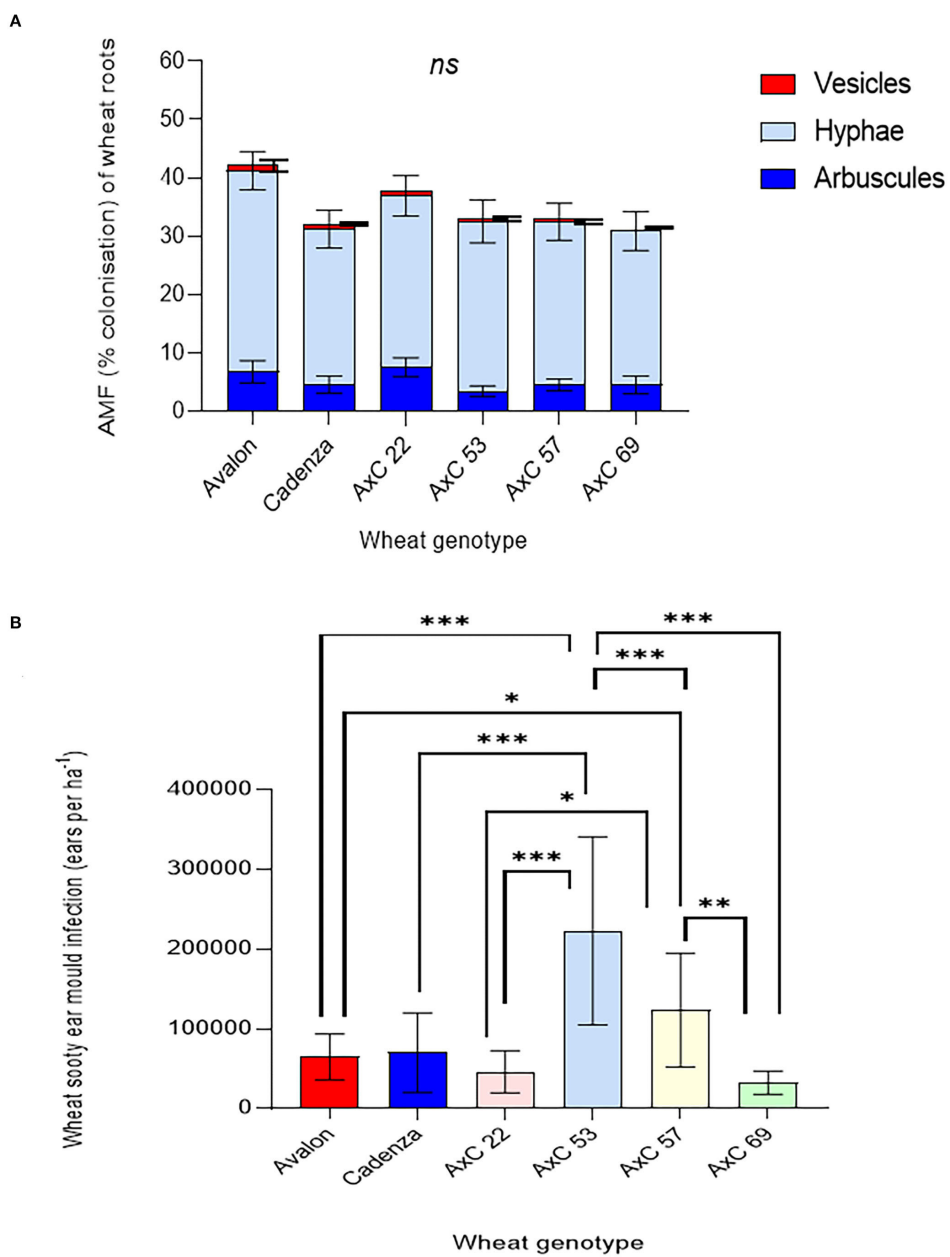
Genotype effects of wheat lines Avalon, Cadenza, AxC22, AxC53, AxC57, and AxC69 on **(A)** Mycorrhizal abundance (% colonisation of wheat roots by hyphae, arbuscules, and vesicles) and **(B)** Sooty ear mould (infected ears ha^−1^) (*n* = 20 for each wheat genotype). The error bars show ± 1 SE. Results of Tukey's HSD test results of significant differences are shown on the graphs (*p = *0.05;* **p = *0.001;* ***p < *0.0001*).

Although the parental and progeny wheat lines were selected originally based on preliminary findings that suggested contrasting phenotypic responses in mycorrhization, these were not supported in the present study, as we found no significant genotype effects on the extent of the mycorrhiza colonisation (Figure 7A). Indeed, contrary to the preliminary data, Avalon was the best, not worst, performing line for hosting mycorrhiza.

#### Wheat genotype effects on sooty ear mould

There were large genotypic differences in sooty ear mould disease (Figure 7B). AxC 53 was the most infected, with a mean of 22 ears m^−2^, contrasting with the least infected, AxC 69, which had 85% fewer mouldy ears (3.3 m^−2)^ a highly significant difference (*p* < 0.0001). AxC 22 had 79.4% less infection than AxC 53; again, this effect is significant (*p* < 0.0001). Both parent lines (Avalon and Cadenza) had similar infection rates (6.5 ears m^−2^ and 7 ears m^−2^, respectively), which were 68–70% less than AxC 53. While AxC 57 had a 77% increase in disease prevalence when compared with parent line Cadenza (Figure 7B), it had just under 50% of the infection rate of AxC 53 (*p* < 0.0001).

### Interactive effects of genotype, tillage type, and ley on wheat performance

**Figure 8:**
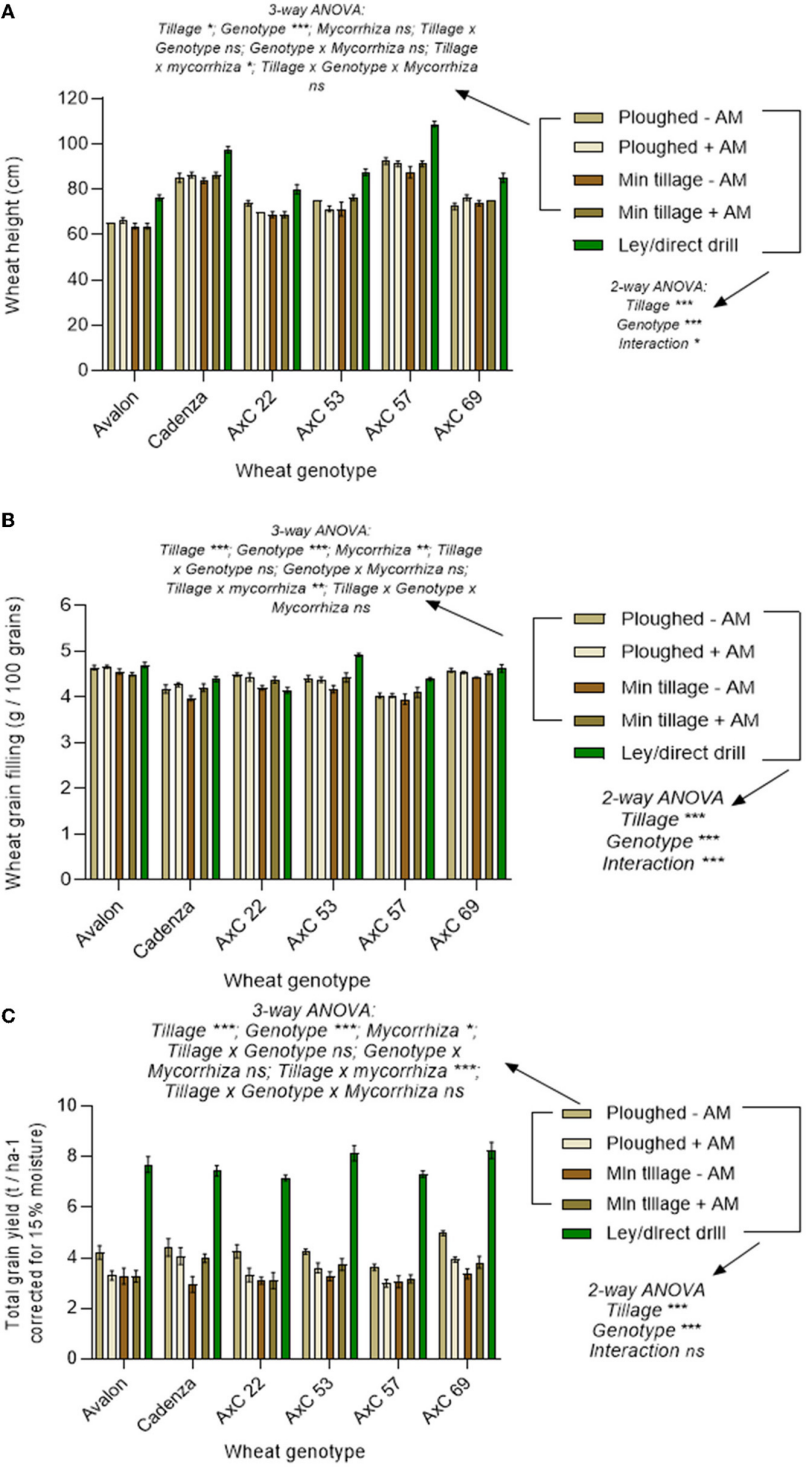
Cultivation and genotype effects on wheat yields from 2018 harvest. Wheat lines Avalon, Cadenza, AxC22, AxC53, AxC57, and AxC69 in ploughed and disc-cultivated plots with a sterile carrier (- AM) or mycorrhizal inoculum (+AM) or direct drilled into ley. **(A)** wheat height (cm) (*n* = 20 for each wheat genotype), **(B)** grain weight (g / 100 grains) (*n* = 20 for each wheat genotype), and **(C)** grain yield (15% moisture content correction, t ha^−1^) (*n* = 20 for each wheat genotype). Error bars show ± 1 SE. The results of the three-way ANOVA testing for effects of genotype, mycorrhizal inoculum and tillage (ploughing vs. disc cultivated) and their interactions are presented for the permanent arable plots (excluding the ley). In parallel, the two-way ANOVA results are presented for effects of tillage (direct drilled ley, disc cultivated, and ploughed) and genotype, and their interaction, averaging across the mycorrhizal inoculum treatments, and including the ley plots. In each case, *p = *0.05;* **p = *0.001;* and ***p < *0.0001*.

The effects of tillage type (ploughed, disc, or direct drilled into ley) on the performance of each of the six wheat lines are shown in Figures 8A–C, with associated two-way ANOVA statistics. There were highly significant effects of the three tillage types on the wheat height at harvest *(p* < 0.001), together with highly significant effects of genotype (*p* < 0.0001) and a significant interaction (*p* = *0.0201*) between these variables (Figure 8A; Supplementary Table S1 for statistical results). AxC57 consistently produced the tallest plants, while Avalon was the shortest (Figure 8A). Wheat consistently grew tallest on the direct drilled ley and tended to be shortest on the disc cultivated treatment, but this varied by genotype, and, sometimes, was no different to ploughing.

As the wheat grew tallest on the ley, it is likely that this treatment gives the clearest indication of the genetic control on wheat height, with fewer growth constraints than on the permanent arable land, on which wheat was grown for a third year in succession. Comparing genotypic responses only in the ley, there was a 35% difference between the height of Avalon, and AxC 57 (76.25 cm and 108.75 cm, respectively, *p* < *0.0001* in Tukey's *post-hoc* tests). Similarly, there was a 30.5% difference between the heights of AxC 22 and AxC 57, with a mean height of 80 cm for AxC 22 (*p* < 0.0001), and a 24% difference between the heights of AxC 57 and AxC69 (*p* < 0.0001). Both AxC53 and AxC 57 have a 21.7% difference in height (*p* < 0.0001). Although smaller (11.5%), the height difference between parent line Cadenza and AxC 57 is also significant (*p* = *0.0002*).

Excluding the ley results, the full factorial analysis of the permanent arable plots to determine the interactive effects of tillage (ploughing vs. disc cultivation) wheat genotype, and adding mycorrhiza inoculum, using three-way ANOVA showed no effects of mycorrhizal inoculum on wheat height (Figure 8A), except in interaction with tillage (*p* < *0.05*), where inoculum reduced the height of the plants on the ploughed plots but increased it on the disc-cultivated plots. This was the only significant interaction term in the three-way ANOVA.

Grain filling (Figure 8B) analysed by the two-way ANOVA showed significant effects of both tillage type (ploughed, disc, or direct drilled into ley) and wheat variety (*p* < 0.0001), and a significant interaction between these variables (*p* < *0.0001*, see Supplementary Tables S1, S2 for statistical results of ANOVA tests). All genotypes produced the greatest individual seed weights when grown on the ley, except AxC 22. This only produced 4 g per 100 grains, which was the lowest value of all genotypes, and contrasted to 4.9 g per 100 grains achieved by AxC 53 on ley (17% difference, < *0.0001*). A trend for reduced grain filling on disc cultivation, especially without the addition of mycorrhizal inoculum, compared to ploughed treatments was seen in five out of the six genotypes: the one exception being Avalon (Figure 8B). This suggests that the AM inoculum slightly improved grain filling, but only in Cadenza, AxC 53 and AxC 57 did this match or exceed the values seen in the uninoculated ploughed treatments.

Three-way ANOVA analysis of the permanent arable plots (excluding the ley) to determine the interactive effects on grain filling of tillage (ploughing vs. disc cultivation) wheat genotype and adding mycorrhiza inoculum (Figure 8B), showed a significant overall increase in grain filling due to mycorrhizal inoculum (*p* < 0.01). This was due to increased grain filling in the mycorrhizal inoculated disc cultivated plots, as in the ploughed plots the grain filling tended to be unresponsive to mycorrhizal inoculation, giving a significant interaction effect between tillage and inoculation and treatments (*p* < 0.01). This was the only significant interaction term in the ANOVA.

Tillage type (ploughed, disc, or direct drilled into ley) and genotype both had significant effects on total grain yields (*p* < 0.0001), but there was no interaction between these variables (*p* > *0.05*), as the different genotypes showed very consistent treatment responses (Figure 8C). As the main effects of tillage type and AMF inoculation on yields have been described previously (Figure 4C), here we focus on the genotype responses. Previously we showed no effect of genotype on yields, averaging across all treatments (Figure 6C), but this analysis gave very high variance because of the differences in yields between the ley and long-term arable treatments. Therefore, focusing on genotype effects on yields on the ley (Figure 8C), AxC 69 produced the highest yield of 8.3 t ha^−1^, with AxC 53 in second place with 8.2 t ha^−1^, and AxC 22 giving the lowest yield of 7.2 t ha^−1^,, but none of these differences are significant (*p* > 0.05). AxC 69 also gave the highest yield (5 t ha^−1^) on the ploughed treatment without AMF inoculation, compared to only 3.6 t ha^−1^ achieved by AxC 57. Yields on the ploughed and disc-cultivated plots, with and without AMF inoculation, varied no more than 1.1 t ha^−1^ between genotypes.

Three-way ANOVA of the permanent arable plots (excluding the ley) to determine the interactive effects on grain yield of tillage (ploughing vs. disc cultivation) wheat genotype, and adding mycorrhiza inoculum (Figure 8C), showed a significant overall effect of mycorrhizal inoculation (*p*<*0.05*), which reduced yields, and a more significant interaction effect between tillage and mycorrhizal treatments (*p*<*0.001*) arising from the average yield loss from mycorrhizal inoculum applied to ploughed plots of 0.75 t ha^−^1, compared to the average gain of 0.37 t ha^−1^ in the disc-cultivated plots receiving inoculum.

#### Effects of tillage/ley, and genotype on mycorrhizal colonisation of wheat

**Figure 9:**
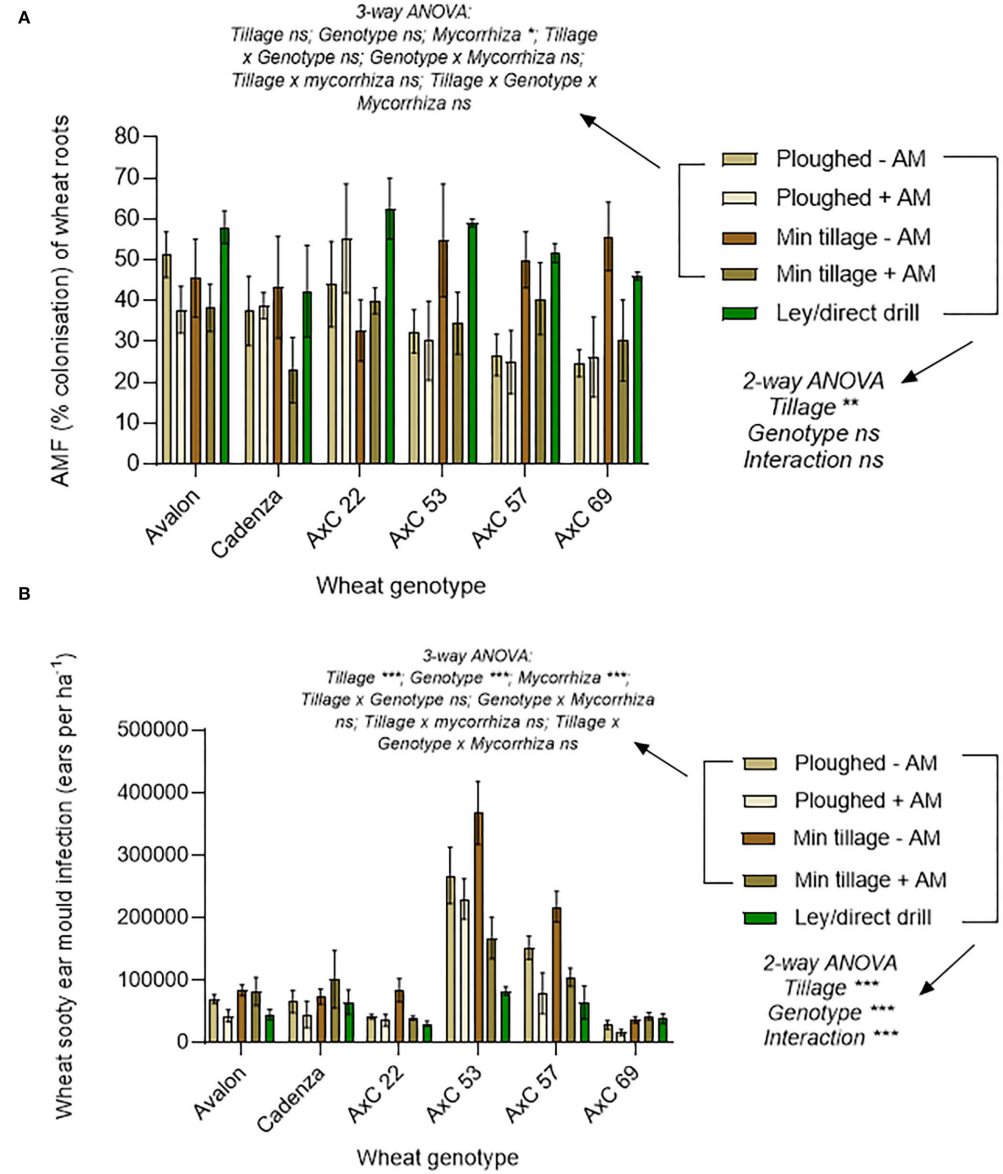
Effects of wheat genotype (Avalon, Cadenza, AxC22, AxC53, AxC57, AxC69), tillage (plough, disc cultivated, and direct drill), mycorrhiza inoculum (- sterile carrier, + commercial mycorrhizal inoculum), and ley on **(A)** total mycorrhizal abundance (% colonisation of wheat roots by hyphae, arbuscules, and vesicles) (*n* = 16–18 for each wheat line or parent), and **(B)** effects on the amount of sooty ear mould (ears ha^−1^) (*n* = 20 for each wheat line or parent). Error bars show ± 1 SE. The results of the three-way ANOVA testing for effects of genotype, mycorrhizal inoculum and tillage (ploughing vs. disc cultivated), and their interactions are presented for the permanent arable plots (excluding the ley). In parallel, the two-way ANOVA results are presented for effects of tillage (direct drilled ley, disc cultivated, and ploughed) and genotype, and their interaction, averaging across the mycorrhizal inoculum treatments, and including the ley plots. In each case, *p = *0.05;* **p = *0.001;* and ***p < * 0.0001*.

There was no significant effect of genotype nor any interaction between genotype and tillage type (ploughed, disc, or direct drilled into ley) on AMF colonisation of wheat roots, as shown by a two-way ANOVA. This is, in part, due to a high variability between replicates (Figure 9A), but there were significant effects of tillage type (*p* = *0.016*), which were attributable to the effects of the ley. This was shown in the three-way ANOVA analysis of the permanent arable plots, excluding the ley, where tillage had no significant effect, and there were no significant interactions between tillage, genotype, and mycorrhizal inoculation. There was a significant overall effect of mycorrhiza inoculum in this analysis (*p* < *0.05*) because of a decrease in mean mycorrhizal colonisation of roots with inoculum addition. See Supplementary Table S1 for full statistical results and Supplementary Figure S3 for a breakdown of hyphae, vesicles, and arbuscules for each wheat genotype and land use type.

In four out of six genotypes, mycorrhizal colonisation was highest when the seeds were direct drilled into former ley, but in two cases (AxC 69 and Cadenza) mycorrhization was highest in the disc-cultivated plots with no mycorrhiza inoculum added, and this latter treatment gave the second highest colonisation in AxC 53 and AxC 57. Importantly, except for AxC 22, there was no evidence that AMF inoculation enhanced mycorrhizal colonisation of any wheat genotype, and, in most cases, contrarily showed a tendency to decrease colonisation, especially in the disc-cultivated plots, where it fell by 7.25% in Avalon to 25.5% in AxC 69. The exception, AxC 22, showed root colonisation increased by AMF inoculation by 7.25% in the disc-cultivated plots and 11.25% in the ploughed, but these were not significant increases. Both Avalon and AxC 22 showed surprisingly high rates of mycorrhizal colonisation in ploughed plots, compared to the other wheat genotypes and other treatments.

Overall, the selection of the parental lines and their offspring genotypes for inclusion in this study based on varying mycorrhizal competency determined in previous unpublished work is not supported by these data. The overall trends reiterated those seen in both Figure 5A, 7A, where Cadenza and AxC 69 showed the least amount of mycorrhiza, and the cultivated soils where no inoculum was added show the greatest amounts of colonisation in the long-term arable plots.

#### Effects of tillage/ley and genotype on sooty ear mould

Sooty ear mould infection was significantly affected by tillage type (ploughed, disc, or direct drilled into ley), wheat genotype, and their interactions (*p* < *0.001*, in each case in the two-way ANOVA; Figure 9B). Two genotypes (AxC 53 and AxC 57) showed particularly high susceptibility to the disease, while AxC 69 showed the highest resistance. Low rates of disease were found in the ley treatment, with this providing the lowest infection rate in Avalon, AxC 22, AxC 53, and AxC 57. The highest rates of infection were seen in the disc cultivated control treatment without AMF inoculum in AxC 53, AxC 57; AxC 22 and Avalon. The addition of AMF inoculum to the disc-cultivated treatment halved infection rates in AxC 53, AxC 57, and AxC 22, but had no effect with Avalon, Cadenza, and AxC 69. AMF inoculation consistently reduced sooty ear mould in ploughed plots with all six wheat genotypes. In Avalon, Cadenza, and AxC 69, disease prevalence, while low, was greater in the ley than in the ploughed soils with added inoculum.

The three-way ANOVA of the permanent arable plots (excluding the ley) to determine the interactive effects on sooty ear mould of tillage (ploughing vs. disc cultivation) wheat genotype, and adding mycorrhiza inoculum, revealed no interaction effects but showed significant effects of all three treatments (Figure 9B).

## Discussion

Conventionally grown high-input wheat has been the main crop in large areas of arable land in the UK and western Europe for several decades, typically covering over 20 million ha (Eurostat, 2021). The main method of cultivation has been annual ploughing and using short rotations with high N fertiliser inputs. This system is increasingly recognised as being both economically and environmentally unsustainable (Agrii, 2016; Goucher et al., 2017; Defra, 2019). Such intensive production degrades soil quality and functions, increasing wheat susceptibility to flood and drought stress (Berdeni et al., 2021), and pathogens (Zetzsche et al., 2020), while adversely impacting beneficial soil biota (Helgason et al., 1998; Oehl et al., 2010; Prendergast-Miller et al., 2021). The increasing interest in regenerative agriculture and farming methods that, in contrast, seek to work with, rather than against, beneficial organisms (LaCanne and Lundgren, 2018; Newton et al., 2020; Schreefel et al., 2020) require a better understanding of the effects of management interventions on these organisms, such as mycorrhizas. The impetus for substantive changes to intensive arable farming practises to reduce inputs has strengthened with the escalating costs of fertilisers (Baffes and Koh, 2021), for which demand can be reduced by including legumes in arable rotations (Berge et al., 2016; McKenna et al., 2018), and diesel fuel use, which can be reduced by adopting no-tillage cultivation (Holland, 2004). Furthermore, in the UK, the replacement of the Common Agricultural Policy of the European Union, with a new farm payment system based on the principle of “public money for public goods” prioritises biodiversity, soil health, and reducing water and air pollution, to include payments for introducing legume-rich multispecies leys in arable rotations (Defra, 2021b). In this scientific and policy context, our multifactorial field trial tested four hypotheses about the effects on yields and ear pathology of mycorrhizal inoculants, reducing tillage, reintroduction of grass-clover leys followed by direct drilling, for six wheat genotypes planted on intensively cultivated and cropped arable land, providing timely and important new information.

Contrary to our first hypothesis, the addition of a commercial mixed species, UK-sourced mycorrhiza inoculum to replicated intensively managed arable field plots failed to increase either wheat mycorrhization or yield, contrasting with some previous studies in other countries with less intensive production systems that have shown these benefits (Pellegrino et al., 2015; Ramadas et al., 2019). Our findings are partly consistent with the pot-based study of the effects of AMF inoculation of wheat grown in UK arable soil by Elliott et al. (2021), who found no benefit to crop mineral nutrition, despite, in that case, enhanced mycorrhization with inoculation. In this study, AMF inoculum reduced ear disease incidence in the more susceptible crop genotypes, but since it did not increase mycorrhization, this effect is more likely to be caused by bacteria, such as Pseudomonads, that routinely co-associate with pot-grown AMF inoculum (Roesti et al., 2005; Cruz and Ishii, 2012; Agnolucci et al., 2015). Previous studies have shown such root and AMF associated-bacteria can induce systemic host defences (Behn, 2008) and reduce pathogen infections (Cruz and Ishii, 2012; Pérez-de-Luque et al., 2017). Fluorescent Pseudomonads are particularly effective in reducing wheat ear mould mycotoxin production by *Alternaria* (Müller et al., 2018). Furthermore, the reduced ear pathology involved a yield penalty, likely due to the physiological costs of inducing systemic resistance to pathogens (Cameron et al., 2013), so from a farmer's perspective the AMF inoculum had net costs with no economic benefits, apart from a likely reduction in mycotoxins, which we did not measure.

Contrary to our second hypothesis, because we found no yield gains from the AMF inoculum, there was no evidence that the yields in ploughed plots benefitted more from inoculum than the disc-cultivated plot. However, the reduction in ear pathology due to AMF inoculum was unexpectedly greater for the disc-cultivated plots, an effect that was driven mainly by the higher rates of sooty ear mould in uninoculated disc-cultivated plots, likely due to greater infection from the unburied crop residues from the previous year, compared to the ploughed treatment. Such effects have previously been shown for *Fusarium* head blight in wheat, which generates the mycotoxin deoxynivalenol that is more abundant in wheat grain harvested from non-inversion tillage than ploughed plots, in a study lasting more than a decade (Morris, 2016).

Our third hypothesis that 3-year grass-clover leys regenerate soil biology and build fertility, giving higher mycorrhization, improved yields, and reduced N fertiliser requirement was strongly supported. However, the prediction that shoot pathology would be worse due to the possible transfer of pathogens from residues of the ley species was not supported- indeed wheat shoot health appeared enhanced by the former ley, which may be due to the break from cereal cropping for 3 years. It could also arise from enhanced induced resistance through interactions with beneficial microorganisms, including the relatively high rates of mycorrhiza colonisation (Cameron et al., 2013).

Our fourth hypothesis regarding the effects of wheat genotypes on mycorrhization, and the interaction between variables to give genotype-specific outcomes in relation to the previous three hypotheses, was only partially supported. There were no significant genotype effects on mycorrhization, despite our selection of genotypes that in previous unpublished field trials appeared to differ in mycorrhization. However, there were significant genotype interaction effects with tillage/ley treatments in relation to wheat height, grain filling, and ear pathology. Several studies have shown evidence of genotypic control of mycorrhization in wheat (Hetrick et al., 1995; Lehnert et al., 2017, 2018), but it remains unclear how stable this trait is to different edaphic and management conditions, and if genotypic effects are specific to different AMF species. The likelihood of genotype x environment x mycorrhiza species interactions controlling AMF colonisation, paralleling genotype x environment x management effects on nitrogen use efficiency in wheat traits (Hawkesford and Riche, 2020) is a potentially major challenge to establishing genotypic selection criteria to enhance the benefits of mycorrhiza to wheat under field conditions.

Importantly, our research shows that the addition of commercial mycorrhizal inoculum to intensively cultivated UK arable soil is no substitute for reintroducing legume-rich leys into arable rotations to build soil fertility and mycorrhizal inoculum that is locally adapted and able to colonise wheat roots.

### Key findings: Wheat performance improved on direct drilling into a 3-year ley

Wheat yields achieved after glyphosate treatment and direct drilling into a 3-year mown grass-clover ley, simulating silage cutting, were comparable to current UK yields (~7 t ha^−1^) (Defra, 2020), using barely a quarter (35 kg ha^−1)^ of the average N application rate of ~137 kg ha^−1^ (Defra, 2018). Our rate of fertiliser application is comparable to annual applications of farm yard manure (organic fertiliser) that were applied regularly to arable soils until the 1960–1970s (Johnston and Poulton, 2018), and achieves very high nitrogen use efficiency with very low losses compared to conventional intensive high-input production (Hawkesford, 2014). The wheat yields obtained on the ley compare very favourably with those of organically grown wheat in the UK. These typically range from 2 to 5 t ha^−1^, exceptionally exceeding 7 t ha^−1^ after soil fertility building using 2-year clover-rich leys mown and mulched, and additions of green waste composts or animal manures, including chicken manure (Stanley and Wilcockson, 2010). Our use of herbicide and slug pellets, and low rates of N fertiliser, but no seed treatment chemicals, fungicides, or insecticides, is distinct from both conventional and organic farming practises. The yields we obtained should be interpreted in relation to the substantially reduced chemical inputs (and associated costs) relative to conventional arable farming to evaluate the economic and environmental benefits of reintroducing leys into arable rotations.

We did not apply any growth regulators to the wheat, and Cadenza and many of the Avalon x Cadenza double haploid lines are taller than modern high-yielding short-stemmed wheat with dwarfing genes like Avalon, which have been bred to reduce the risks of crop lodging in windy and wet weather. Consequently, when grown on the former ley, wheat height was generally taller than average for modern varieties under intensive management shown in the Wheat Growth Guide (AHDB, 2021), while the third year of wheat growing in the long-term arable plots showed dwarfing likely induced by nitrogen limitation. This was also reflected in the poorer grain filling compared to the wheat direct drilled into the ley and corroborates the improved grain filling seen after simulated direct drilling into a 19-month grass-clover ley in a mesocosm study on the same soils as this study (Berdeni et al., 2021). Furthermore, the latter study showed major improvements in earthworm numbers, soil structure (reduced bulk density), and hydrological functioning (increased water-holding capacity, infiltration rates, and saturated hydraulic conductivity) in 19-month-old grass-clover leys compared to long-term arable soil, improving wheat growth and yields under moderate drought and flooding. In the present study, the spring of 2018 was unusually dry, and the likely increase in soil pore space in the ley generated by root channels from the perennial grass and tap-rooted clover plants likely contributed to the wheat yields matching and exceeding national average values despite the low rate of fertiliser use in the study.

As expected, AMF colonisation of wheat roots was higher when direct drilled into the 3-year ley than in the long-term arable plots, since the ley soil was undisturbed for more than 3 years, and mycorrhizas will have been supported all year round by the evergreen leys. This is likely to support a large active mycelial network that would improve nutrient exchange and plant performance (Manoharan et al., 2017), especially as the herbicide treatment did not completely kill all of the clover. The effects of the herbicide on mycorrhiza in our study remain unresolved, but had the ley been terminated without herbicide or soil disturbance such as by mowing very short, mycorrhization may have been even greater given the sometimes-reported adverse effects of glyphosate (Druille et al., 2013a,b). Nonetheless, the combination of glyphosate and direct drilling into ley, with some clover plants surviving, resulted in significantly higher mycorrhization than conventional intensive arable cropping. Improved mycorrhization in the ley is also likely to have improved soil macroaggregation, soil structure, and water retention as shown previously for AMF (Wilson et al., 2009; Gianinazzi et al., 2010), and therefore assist the soil regeneration reflected in the yields achieved on the former ley.

Overall, the benefits we have found from the inclusion of grass-clover leys in arable rotations indicate that this should be promoted in farming policies that are concerned with reducing environmental pollution and enhancing biodiversity, which are the stated objectives of the UK government Environmental Land Management scheme (Defra, 2021a). The benefits from these leys include the evidence from the present study of reduced need for N fertiliser with its large environmental impacts (Goucher et al., 2017), improving functional biodiversity including mycorrhizas, as shown here, and earthworms (Berdeni et al., 2021; Prendergast-Miller et al., 2021). These organisms enhance soil health in leys by enhancing infiltration rates and soil water storage capacity as recently shown, in adjacent fields to the present study, at Leeds University farm (Hallam et al., 2020; Berdeni et al., 2021), and increase soil C sequestration and soil aggregate stability. Increased carbon sequestration and improved hydrological functioning, which reduces flood, soil erosion, and water pollution risks, together with improved functional biodiversity from reintroducing leys into arable rotations deliver multiple ecosystem service benefits. Nonetheless, it remains unclear if 3-year grass-clover leys are the best management system for sustainably improving soil health, crop, and livestock production. A comprehensive review of the potential benefits of species-rich herbal leys containing legumes and deep-rooted grasses suggests that these may be preferable particularly for livestock grazing, as antihelminthic compounds in some herbs improve livestock health and productivity (Cooledge et al., 2022). Furthermore, herbal leys can help to reduce greenhouse gas emissions from enteric methane production by ruminant livestock and nitrous oxide emissions from urine patches, since the herbage chemistry can reduce methanogenesis, and high tannin content results in more dietary N ending in dung and less in urine (Cooledge et al., 2022). Such diverse herbal leys are now actively promoted in the UK by subsidy payments (Defra, 2021a) but their effects on soil health, including organic carbon, earthworms, mycorrhizas, soil aggregation, hydrological functioning, and subsequent crop yields relative to grass-clover leys, remain to be established.

### Key findings: Commercial mycorrhizal inoculum reduced mycorrhization and wheat yields after ploughing and gave modest yield gains with disc cultivation

In contrast to the dual benefits of increased mycorrhization and wheat yields seen in the ley, the addition of commercial mixed species AMF inoculum of UK provenance consistently reduced yields in ploughed plots and gave variable, but only modest yield gains under direct drilling that were not significant in 2018, and was associated with reduced mycorrhization in both tillage treatments. These findings were contrary both to our expectations and some previous research in other countries (Pellegrino et al., 2015). However, Salomon et al. (2022) evaluated 25 commercial mycorrhizal inoculants sold in Australia, Europe, and North America in pot trials and concluded that only one product increased mycorrhizal colonisation of highly mycorrhiza-responsive plants (*Lycopersicum esculentum* and *Allium ameloprasum*) in non-sterile soil, and highlighted the need for more rigorous quality control of these products and more field trials. Pot experiments using arable soils with the addition of a single species commercial AMF inoculum (*Rhizophagus irregularis*) have shown increases in wheat root mycorrhiza colonisation but had little, or even negative effects on macronutrient uptake and concentrations in wheat grains (Elliott et al., 2021; Watts-Williams and Gilbert, 2021), suggesting that mycorrhiza may often play a limited role in wheat nutrition in long-term arable soils. AMF are, normally, functionally most important for plant P nutrition. The limited mycorrhization and lack of positive yield response to inoculation may be due to P not being limited to these plants, likely due to historical enrichment from decades of high fertiliser use, compounded by N limitation from the low rates of fertiliser use in our trial. This will constrain plant P demand and any benefit of mycorrhiza for P nutrition. In addition, P mobilising bacteria in the mycorrhizal inoculum could potentially enhance P supply to the wheat and lead to suppression of mycorrhizal colonisation. In contrast, in the direct drilled legume-rich ley, symbiotically fixed N enabled greater wheat biomass production and associated demands for other nutrients. In the ley, the benefits of AMF for P nutrition are therefore likely to be enhanced, especially with organic P being released from the senescing ley herbage and roots following the glyphosate treatment (Eason et al., 1991).

Since the AMF inoculum in our studies did elicit strong disease suppressive effects, particularly in the disc-cultivated plots, it seems likely that the yield penalty arising from the use of the inoculum arose from the induction of systemic resistance to pathogens (Mustafa et al., 2017). What is unclear is whether it was AMF fungi in the inoculum that elicited this response, or other microorganisms, such as Pseudomonads that are likely to be co-cultured in commercially produced mycorrhizal inoculum produced from pot-cultured plants (Roesti et al., 2005; Agnolucci et al., 2015). Rhizobacteria such as Pseudomonads can similarly induce systemic defences in plants, and can also interact with AM in this process to inhibit pathogens as shown in wheat (Behn, 2008; Pérez-de-Luque et al., 2017). If the systemic resistance to pathogens was induced by microorganisms other than mycorrhizal fungi, this might also help explain why the AMF inoculum tended to reduce rather than increase mycorrhizal colonisation of roots. The defense reactions potentially may have inhibited colonisation by native AMF in the soil, and the metabolic costs of defences may have restricted carbon allocation to mycorrhiza that did manage to colonise roots.

Other studies have suggested that the benefits of field inoculation may be limited by pre-existing and locally adapted fungal communities outcompeting the introduced strains however, if commercial inoculants are successfully established, they could then have negative impacts on native soil biodiversity and associated plant species (Renaut et al., 2020). This highlights another important benefit of deploying leys to build soil fertility and enhance the functioning and activity of the native, locally adapted, AMF populations. In this study, we do not record AMF species naturally occurring in the soil, and the communities present with and without the addition of inoculum. Furthermore, there is now clear evidence that wheat and other arable crops under conventional intensive cultivation practises host not only Glomeromycotean AMF fungi but also closely related Mucoromycotina root fine endophytes that form similar symbiotic associations with roots but are under-reported as they are not detected using AMF-targeted primers for DNA sequence amplification and analyses (Sinanaj et al., 2021). A recent unpublished study (Guest, 2022), also at Leeds University farm, investigated AMF communities in 3-year grass-clover leys compared to long-term arable cropping in four arable fields. This showed significant increases in AMF diversity in the leys showing the soil restorative nature of growing highly mycorrhizal evergreen species like clovers in rotations. Rather than adding inoculants to improve mycorrhization, it would therefore appear preferable to change field management practises to rebuild locally adapted mycorrhizal communities, for example using leys and minimising soil disturbance to establish following crops, ideally by direct drilling. These changes can potentially reduce fuel and fertiliser use and associated costs and environmental pollution.

Investigations into the genetic control of mycorrhizal competence and responsiveness in wheat remain very incomplete, and the quantitative trait loci (QTLs) that determine this association are not well established (Hohmann and Messmer, 2017; Lehnert et al., 2017; De Vita et al., 2018; Berger and Gutjahr, 2021). However, recent studies have begun to demonstrate genetic links through quantitative trait nucleotides (QTNs) (Ganugi et al., 2021), which are likely to be beneficial to future breeding studies. In this study, we found some genotypic variation in wheat performance in the four doubled haploid lines AxC 22, AxC 53, AxC 57, and AxC 69 and parents Avalon and Cadenza, but no difference in their competence in hosting AMF, which was more strongly controlled by plot management (such as ley with direct drilling) than genotype.

## Conclusion

Grass-clover leys can play a major role in regenerating soil biology and fertility, enabling enhancement of mycorrhiza functioning and enhancing crop yields and suppressing shoot pathogens, likely via systemic induced resistance enhanced by root associations with beneficial microorganisms. We show that in as little as 3 years, grass-clover leys regenerate soil health and fertility with increases in yield, reduced fertiliser and fungicide requirements, and improved wheat mycorrhization and shoot health. This delivers substantial benefits in the reduced use of expensive fertilisers and agrochemicals and the environmental pollution associated with their production and use. These benefits are augmented by improvements in soil organic carbon and soil water-holding capacity in previous studies, and overall provide a strong basis for financial support for arable farmers to reintroduce legume-rich leys into arable rotations to deliver public goods and ecosystem services. Inoculation of arable soils with a commercial AMF mix provided no tangible benefits to farmers or the environment and cannot be recommended in a ploughed arable system, and the benefits to yields and pathology reduction in the disc-cultivated system were very modest. Adding mycorrhizal inoculum is certainly no substitute for the benefits of reintroducing leys into arable rotations. Future studies need to investigate how well diverse herbal leys, that are strongly promoted in the UK by Defra for inclusion in arable rotations, perform relative to grass-clover leys for soil health, mycorrhizal functioning, and subsequent crop production, and to compare the effects of grazing vs. mowing of leys.

## Data availability statement

The raw data supporting the conclusions of this article will be made available by the authors, without undue reservation.

## Author contributions

JL and DC designed the study. ST, NA, DB, MN, TH, and ML carried out the experiments and sample collection, with assistance from JL who planned and managed the harvesting operations. NA analysed the data. NA wrote the first draft with contributions from DC and JL. All authors have read and approved the manuscript.

## Funding

We gratefully acknowledge funding for the consortium project MycoRhizaSoil: Combining wheat genotypes with cultivation methods to facilitate mycorrhizosphere organisms improving soil quality and crop resilience (BB/L026066/1, BB/L026023/1, and BB/L026007/1) funded by BBRSC, NERC, and Defra in the Global Food Security Soil and Rhizosphere Interactions for Sustainable Agri-Ecosystems (GFS-SARISA) call, as part of the Soil Security Program.

## Conflict of interest

Author RS was employed by RAGT Seeds Ltd. Author DB was employed by ADAS Gleadthorpe, RSK Group. The remaining authors declare that the research was conducted in the absence of any commercial or financial relationships that could be construed as a potential conflict of interest.

## Publisher's note

All claims expressed in this article are solely those of the authors and do not necessarily represent those of their affiliated organizations, or those of the publisher, the editors and the reviewers. Any product that may be evaluated in this article, or claim that may be made by its manufacturer, is not guaranteed or endorsed by the publisher.
