# Supplementary material for: Experimental evaluation of biological regeneration of arable soil: The effects of grass-clover leys and arbuscular mycorrhizal inoculants on wheat growth, yield, and shoot pathology
Source: Front Plant Sci. 2022 Aug 24;13:955985. doi: 10.3389/fpls.2022.955985 (PMC9450525; doi:10.3389/fpls.2022.955985)
Supplement: Supplementary file 1 [file Presentation_1.pptx]

## Slide 1
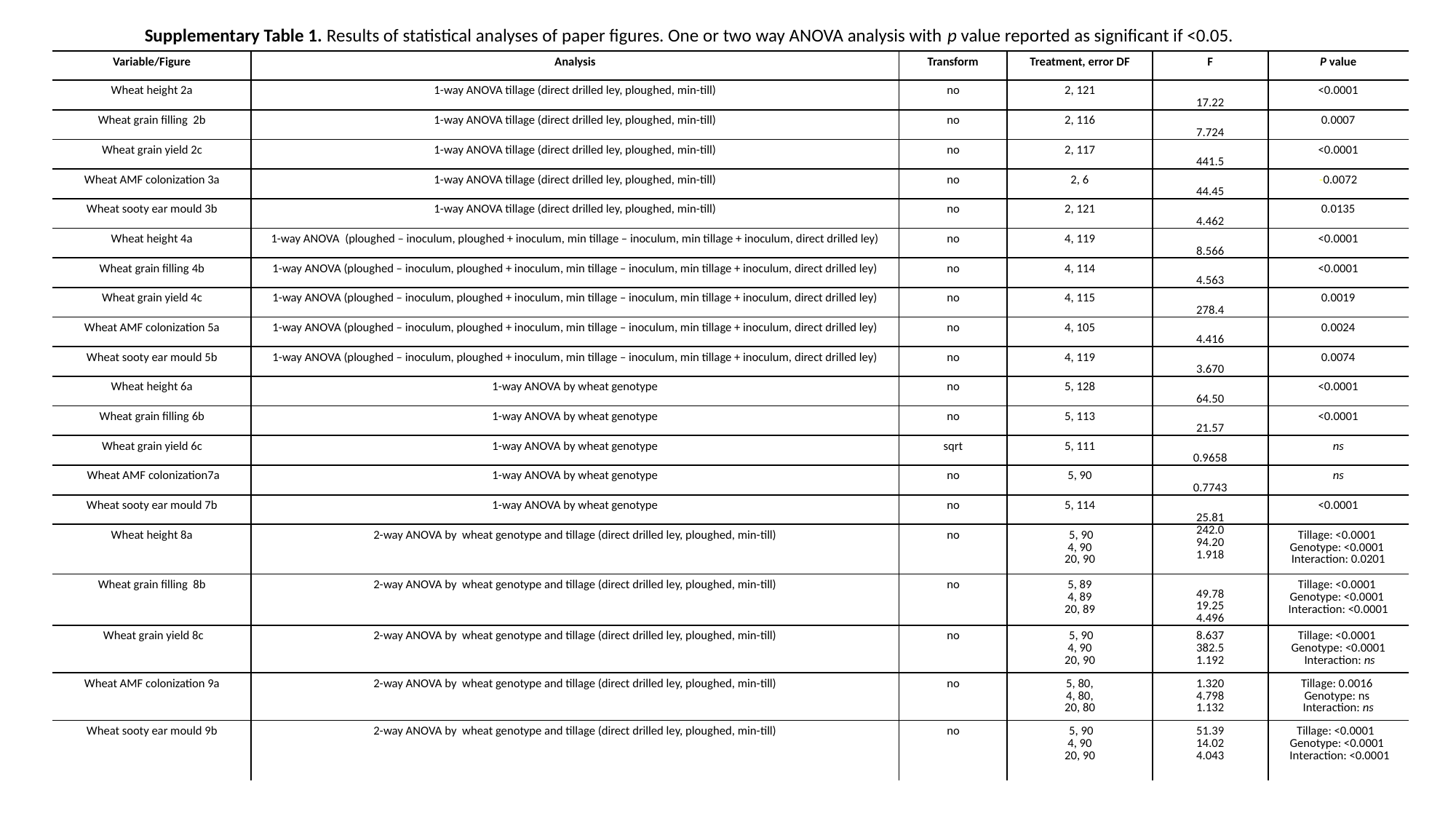

Supplementary Table 1. Results of statistical analyses of paper figures. One or two way ANOVA analysis with p value reported as significant if <0.05.
| Variable/Figure | Analysis | Transform | Treatment, error DF | F | P value |
| --- | --- | --- | --- | --- | --- |
| Wheat height 2a | 1-way ANOVA tillage (direct drilled ley, ploughed, min-till) | no | 2, 121 | 17.22 | <0.0001 |
| Wheat grain filling 2b | 1-way ANOVA tillage (direct drilled ley, ploughed, min-till) | no | 2, 116 | 7.724 | 0.0007 |
| Wheat grain yield 2c | 1-way ANOVA tillage (direct drilled ley, ploughed, min-till) | no | 2, 117 | 441.5 | <0.0001 |
| Wheat AMF colonization 3a | 1-way ANOVA tillage (direct drilled ley, ploughed, min-till) | no | 2, 6 | 44.45 | -0.0072 |
| Wheat sooty ear mould 3b | 1-way ANOVA tillage (direct drilled ley, ploughed, min-till) | no | 2, 121 | 4.462 | 0.0135 |
| Wheat height 4a | 1-way ANOVA (ploughed – inoculum, ploughed + inoculum, min tillage – inoculum, min tillage + inoculum, direct drilled ley) | no | 4, 119 | 8.566 | <0.0001 |
| Wheat grain filling 4b | 1-way ANOVA (ploughed – inoculum, ploughed + inoculum, min tillage – inoculum, min tillage + inoculum, direct drilled ley) | no | 4, 114 | 4.563 | <0.0001 |
| Wheat grain yield 4c | 1-way ANOVA (ploughed – inoculum, ploughed + inoculum, min tillage – inoculum, min tillage + inoculum, direct drilled ley) | no | 4, 115 | 278.4 | 0.0019 |
| Wheat AMF colonization 5a | 1-way ANOVA (ploughed – inoculum, ploughed + inoculum, min tillage – inoculum, min tillage + inoculum, direct drilled ley) | no | 4, 105 | 4.416 | 0.0024 |
| Wheat sooty ear mould 5b | 1-way ANOVA (ploughed – inoculum, ploughed + inoculum, min tillage – inoculum, min tillage + inoculum, direct drilled ley) | no | 4, 119 | 3.670 | 0.0074 |
| Wheat height 6a | 1-way ANOVA by wheat genotype | no | 5, 128 | 64.50 | <0.0001 |
| Wheat grain filling 6b | 1-way ANOVA by wheat genotype | no | 5, 113 | 21.57 | <0.0001 |
| Wheat grain yield 6c | 1-way ANOVA by wheat genotype | sqrt | 5, 111 | 0.9658 | ns |
| Wheat AMF colonization7a | 1-way ANOVA by wheat genotype | no | 5, 90 | 0.7743 | ns |
| Wheat sooty ear mould 7b | 1-way ANOVA by wheat genotype | no | 5, 114 | 25.81 | <0.0001 |
| Wheat height 8a | 2-way ANOVA by wheat genotype and tillage (direct drilled ley, ploughed, min-till) | no | 5, 90 4, 90 20, 90 | 242.0 94.20 1.918 | Tillage: <0.0001 Genotype: <0.0001 Interaction: 0.0201 |
| Wheat grain filling 8b | 2-way ANOVA by wheat genotype and tillage (direct drilled ley, ploughed, min-till) | no | 5, 89 4, 89 20, 89 | 49.78 19.25 4.496 | Tillage: <0.0001 Genotype: <0.0001 Interaction: <0.0001 |
| Wheat grain yield 8c | 2-way ANOVA by wheat genotype and tillage (direct drilled ley, ploughed, min-till) | no | 5, 90 4, 90 20, 90 | 8.637 382.5 1.192 | Tillage: <0.0001 Genotype: <0.0001 Interaction: ns |
| Wheat AMF colonization 9a | 2-way ANOVA by wheat genotype and tillage (direct drilled ley, ploughed, min-till) | no | 5, 80, 4, 80, 20, 80 | 1.320 4.798 1.132 | Tillage: 0.0016 Genotype: ns Interaction: ns |
| Wheat sooty ear mould 9b | 2-way ANOVA by wheat genotype and tillage (direct drilled ley, ploughed, min-till) | no | 5, 90 4, 90 20, 90 | 51.39 14.02 4.043 | Tillage: <0.0001 Genotype: <0.0001 Interaction: <0.0001 |

## Slide 2
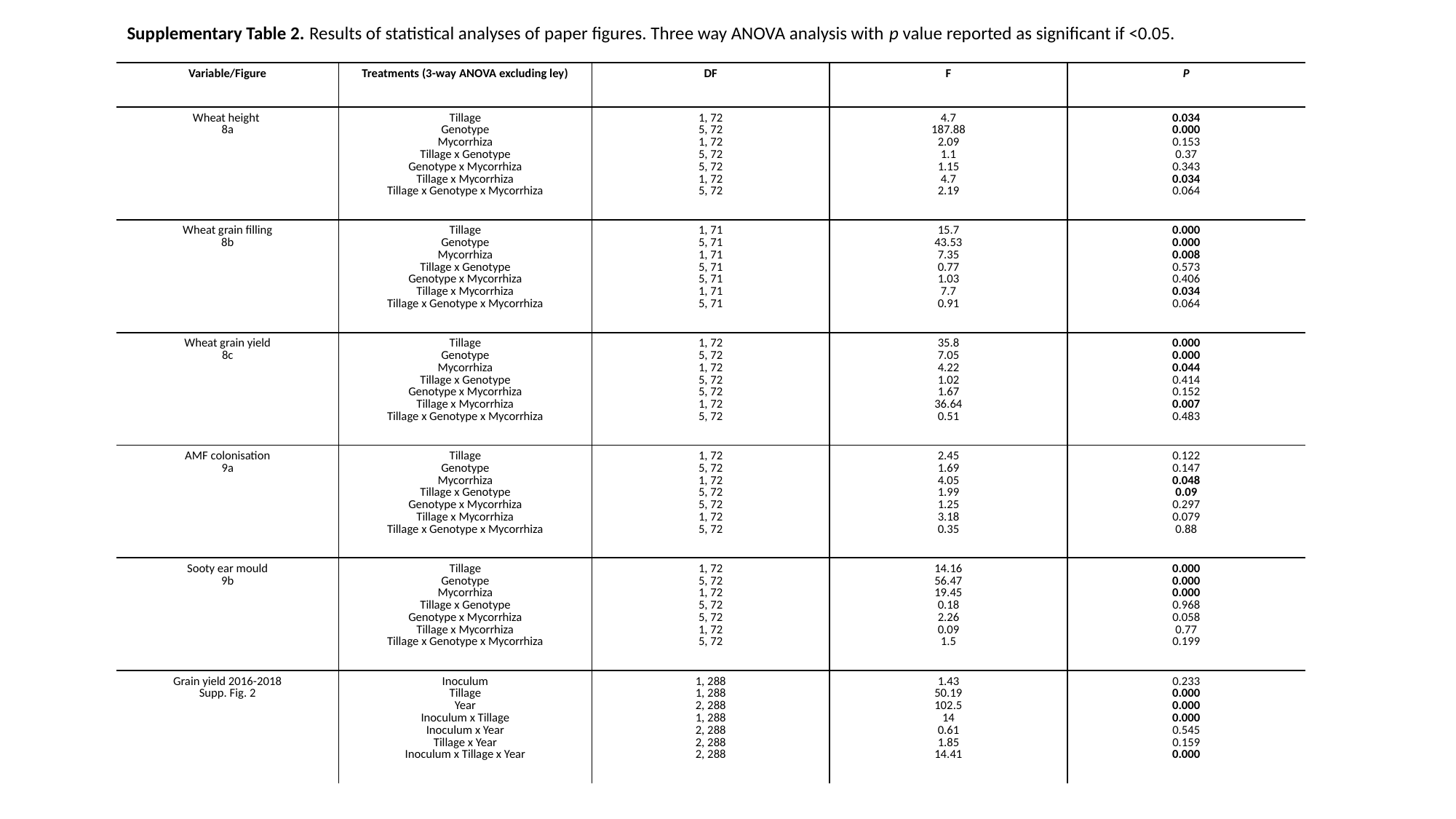

Supplementary Table 2. Results of statistical analyses of paper figures. Three way ANOVA analysis with p value reported as significant if <0.05.
| Variable/Figure | Treatments (3-way ANOVA excluding ley) | DF | F | P |
| --- | --- | --- | --- | --- |
| Wheat height 8a | Tillage Genotype Mycorrhiza Tillage x Genotype Genotype x Mycorrhiza Tillage x Mycorrhiza Tillage x Genotype x Mycorrhiza | 1, 72 5, 72 1, 72 5, 72 5, 72 1, 72 5, 72 | 4.7 187.88 2.09 1.1 1.15 4.7 2.19 | 0.034 0.000 0.153 0.37 0.343 0.034 0.064 |
| Wheat grain filling 8b | Tillage Genotype Mycorrhiza Tillage x Genotype Genotype x Mycorrhiza Tillage x Mycorrhiza Tillage x Genotype x Mycorrhiza | 1, 71 5, 71 1, 71 5, 71 5, 71 1, 71 5, 71 | 15.7 43.53 7.35 0.77 1.03 7.7 0.91 | 0.000 0.000 0.008 0.573 0.406 0.034 0.064 |
| Wheat grain yield 8c | Tillage Genotype Mycorrhiza Tillage x Genotype Genotype x Mycorrhiza Tillage x Mycorrhiza Tillage x Genotype x Mycorrhiza | 1, 72 5, 72 1, 72 5, 72 5, 72 1, 72 5, 72 | 35.8 7.05 4.22 1.02 1.67 36.64 0.51 | 0.000 0.000 0.044 0.414 0.152 0.007 0.483 |
| AMF colonisation 9a | Tillage Genotype Mycorrhiza Tillage x Genotype Genotype x Mycorrhiza Tillage x Mycorrhiza Tillage x Genotype x Mycorrhiza | 1, 72 5, 72 1, 72 5, 72 5, 72 1, 72 5, 72 | 2.45 1.69 4.05 1.99 1.25 3.18 0.35 | 0.122 0.147 0.048 0.09 0.297 0.079 0.88 |
| Sooty ear mould 9b | Tillage Genotype Mycorrhiza Tillage x Genotype Genotype x Mycorrhiza Tillage x Mycorrhiza Tillage x Genotype x Mycorrhiza | 1, 72 5, 72 1, 72 5, 72 5, 72 1, 72 5, 72 | 14.16 56.47 19.45 0.18 2.26 0.09 1.5 | 0.000 0.000 0.000 0.968 0.058 0.77 0.199 |
| Grain yield 2016-2018 Supp. Fig. 2 | Inoculum Tillage Year Inoculum x Tillage Inoculum x Year Tillage x Year Inoculum x Tillage x Year | 1, 288 1, 288 2, 288 1, 288 2, 288 2, 288 2, 288 | 1.43 50.19 102.5 14 0.61 1.85 14.41 | 0.233 0.000 0.000 0.000 0.545 0.159 0.000 |

## Slide 3
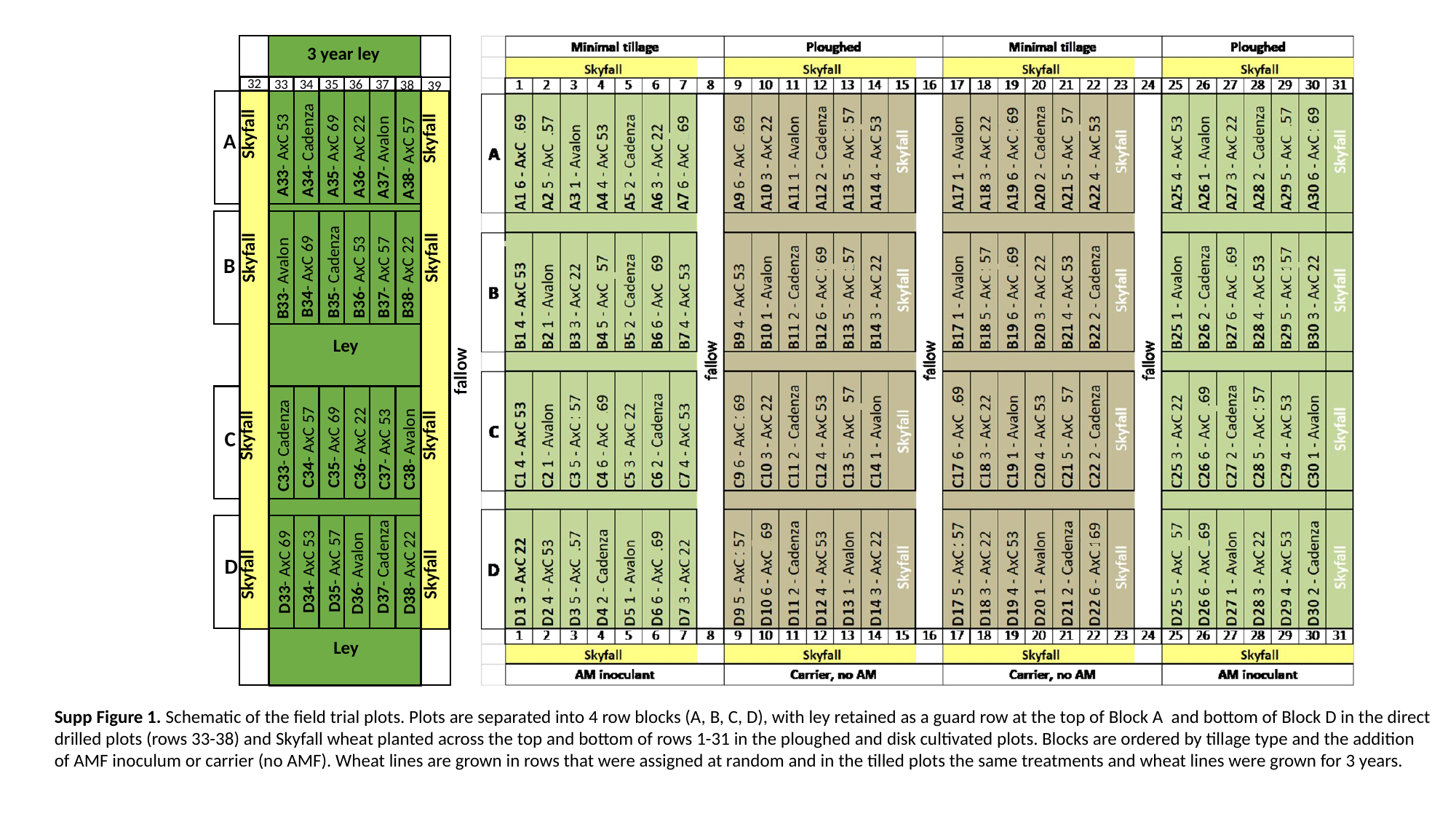

3 year ley
32
34
35
33
37
36
38
39
Skyfall
Skyfall
Skyfall
Skyfall
A33- AxC 53
A34- Cadenza
A35- AxC 69
A36- AxC 22
A37- Avalon
A
A38- AxC 57
fallow
B34- AxC 69
B36- AxC 53
B37- AxC 57
B35- Cadenza
B38- AxC 22
B33- Avalon
B
Skyfall
Skyfall
Ley
C34- AxC 57
C35- AxC 69
C36- AxC 22
C37- AxC 53
C38- Avalon
C33- Cadenza
C
Skyfall
Skyfall
D35- AxC 57
D34- AxC 53
D33- AxC 69
D37- Cadenza
D38- AxC 22
D36- Avalon
D
Ley
Supp Figure 1. Schematic of the field trial plots. Plots are separated into 4 row blocks (A, B, C, D), with ley retained as a guard row at the top of Block A and bottom of Block D in the direct drilled plots (rows 33-38) and Skyfall wheat planted across the top and bottom of rows 1-31 in the ploughed and disk cultivated plots. Blocks are ordered by tillage type and the addition of AMF inoculum or carrier (no AMF). Wheat lines are grown in rows that were assigned at random and in the tilled plots the same treatments and wheat lines were grown for 3 years.

## Slide 4
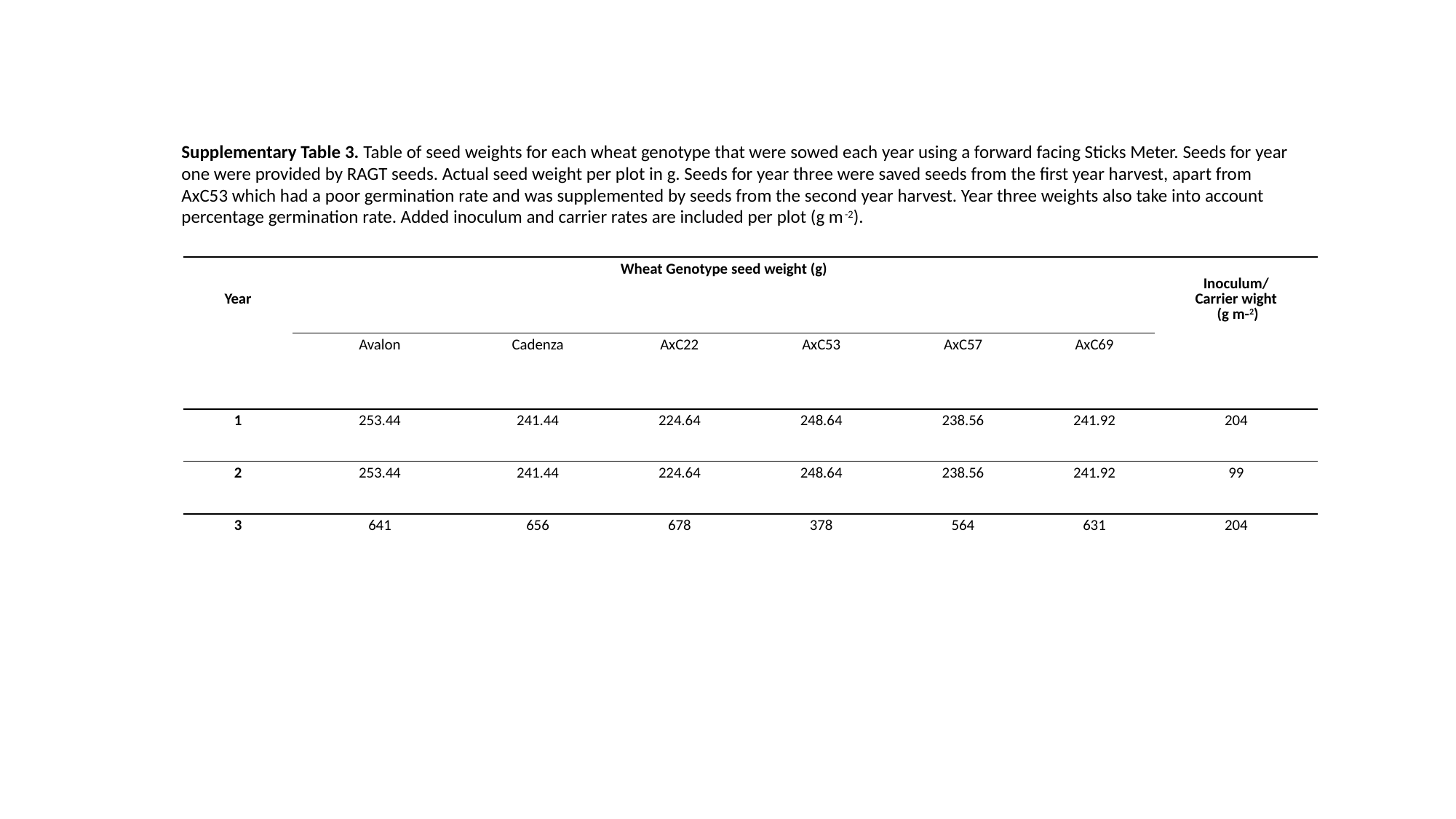

Supplementary Table 3. Table of seed weights for each wheat genotype that were sowed each year using a forward facing Sticks Meter. Seeds for year one were provided by RAGT seeds. Actual seed weight per plot in g. Seeds for year three were saved seeds from the first year harvest, apart from AxC53 which had a poor germination rate and was supplemented by seeds from the second year harvest. Year three weights also take into account percentage germination rate. Added inoculum and carrier rates are included per plot (g m-2).
| Year | Wheat Genotype seed weight (g) | | | | | | Inoculum/ Carrier wight (g m-2) |
| --- | --- | --- | --- | --- | --- | --- | --- |
| | Avalon | Cadenza | AxC22 | AxC53 | AxC57 | AxC69 | |
| 1 | 253.44 | 241.44 | 224.64 | 248.64 | 238.56 | 241.92 | 204 |
| 2 | 253.44 | 241.44 | 224.64 | 248.64 | 238.56 | 241.92 | 99 |
| 3 | 641 | 656 | 678 | 378 | 564 | 631 | 204 |

## Slide 5
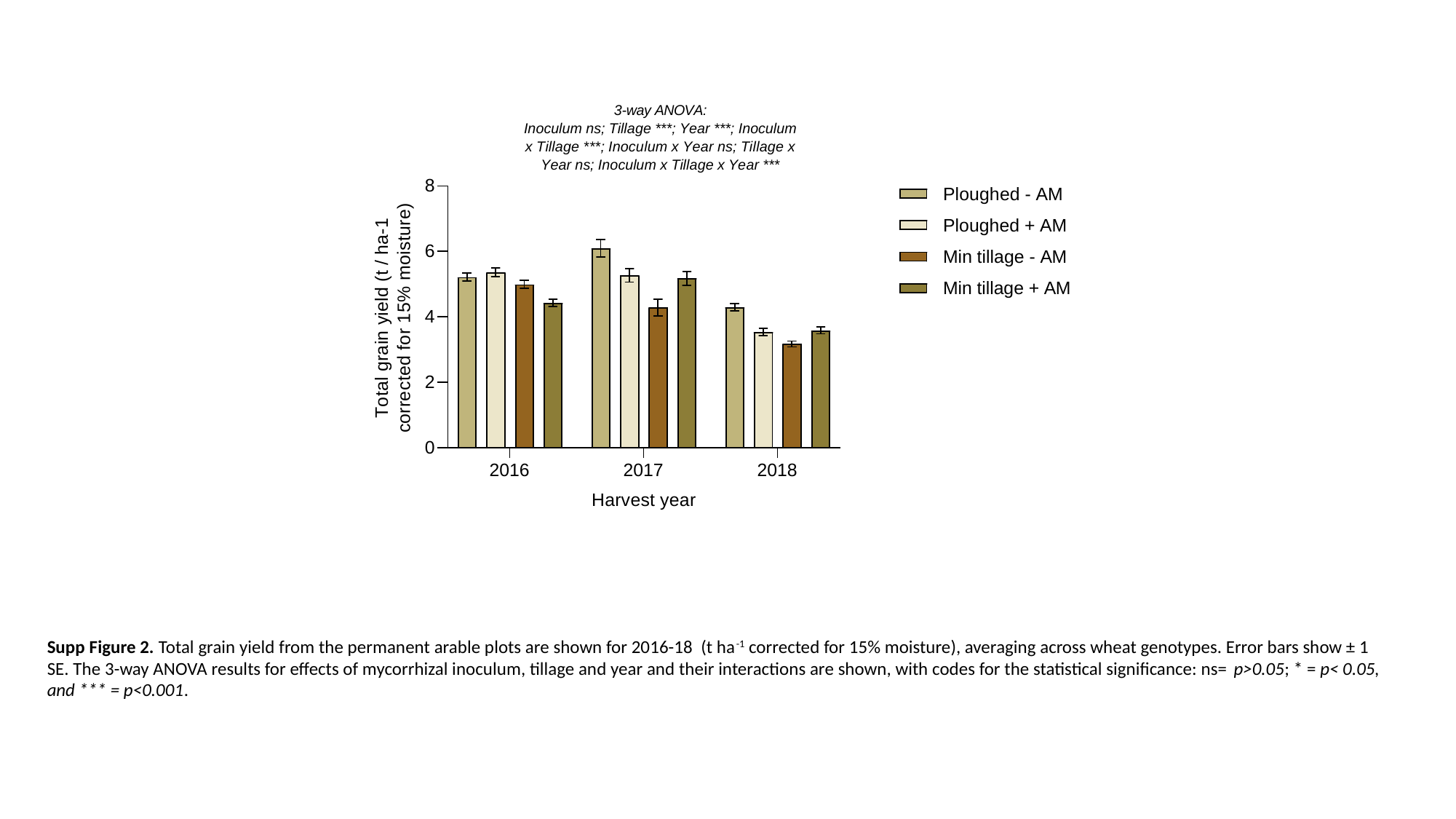

Supp Figure 2. Total grain yield from the permanent arable plots are shown for 2016-18 (t ha-1 corrected for 15% moisture), averaging across wheat genotypes. Error bars show ± 1 SE. The 3-way ANOVA results for effects of mycorrhizal inoculum, tillage and year and their interactions are shown, with codes for the statistical significance: ns= p>0.05; * = p< 0.05, and *** = p<0.001.

## Slide 6
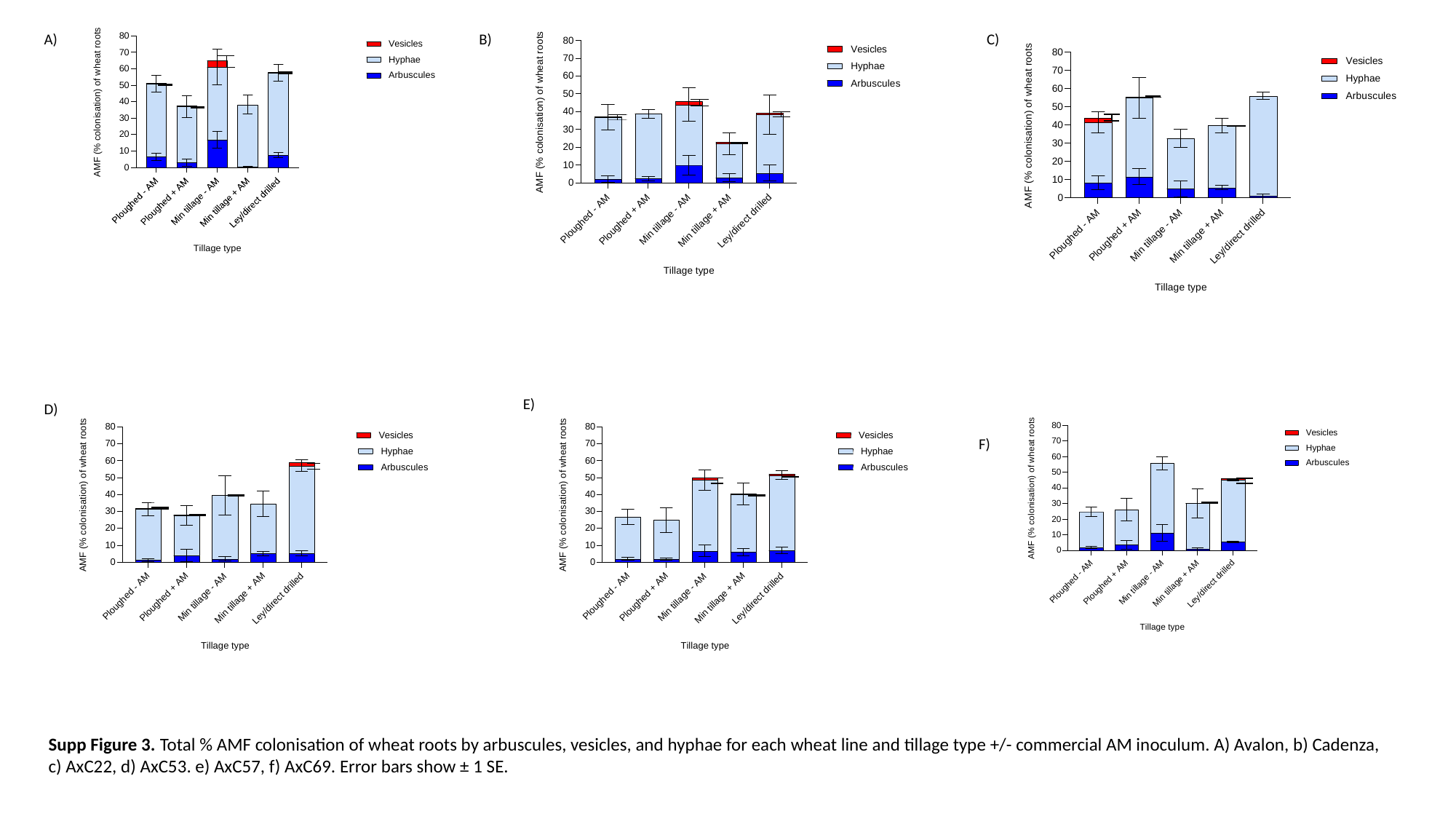

A)
B)
C)
E)
D)
F)
Supp Figure 3. Total % AMF colonisation of wheat roots by arbuscules, vesicles, and hyphae for each wheat line and tillage type +/- commercial AM inoculum. A) Avalon, b) Cadenza, c) AxC22, d) AxC53. e) AxC57, f) AxC69. Error bars show ± 1 SE.

## Slide 7
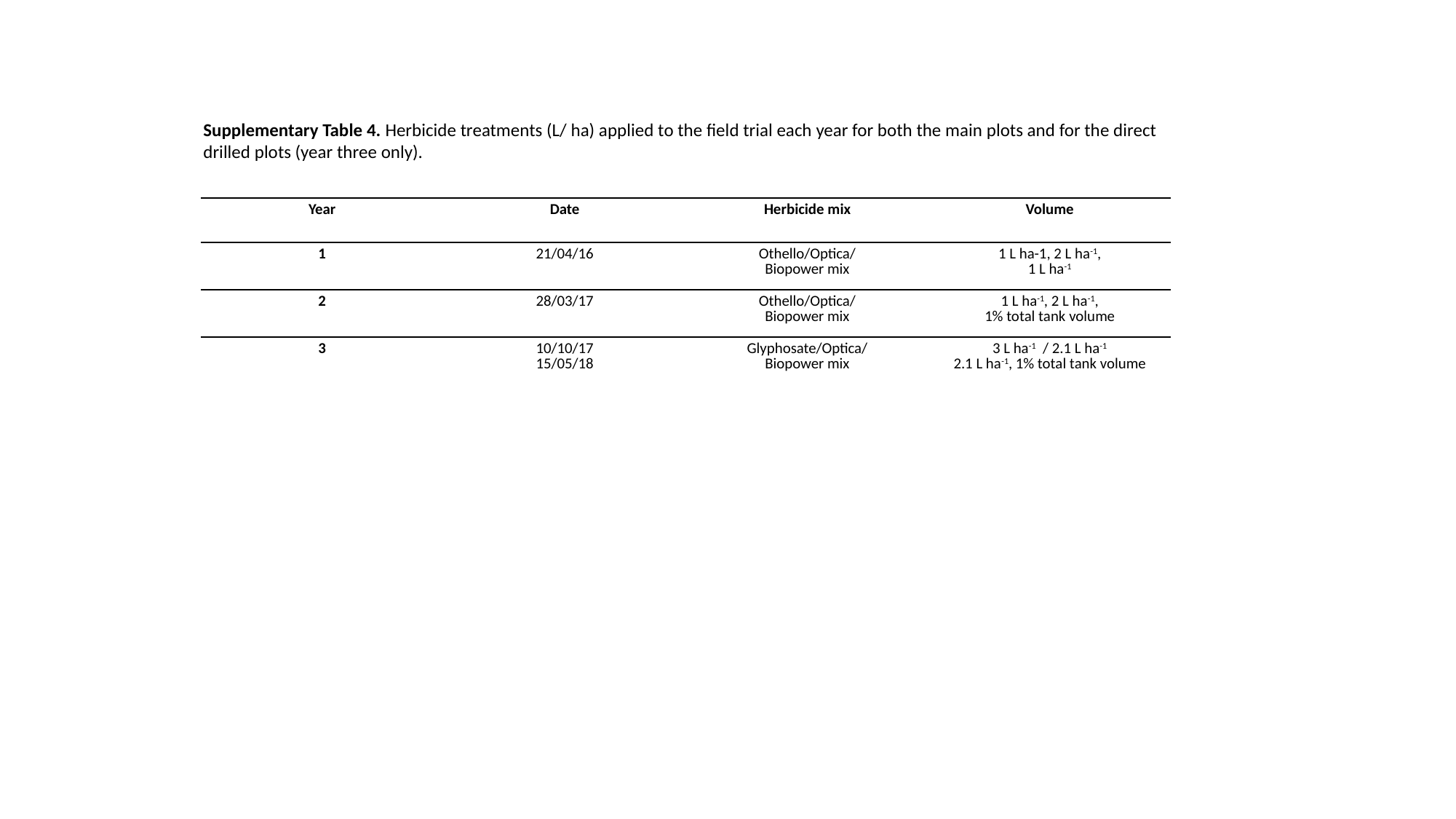

Supplementary Table 4. Herbicide treatments (L/ ha) applied to the field trial each year for both the main plots and for the direct drilled plots (year three only).
| Year | Date | Herbicide mix | Volume |
| --- | --- | --- | --- |
| 1 | 21/04/16 | Othello/Optica/ Biopower mix | 1 L ha-1, 2 L ha-1, 1 L ha-1 |
| 2 | 28/03/17 | Othello/Optica/ Biopower mix | 1 L ha-1, 2 L ha-1, 1% total tank volume |
| 3 | 10/10/17 15/05/18 | Glyphosate/Optica/ Biopower mix | 3 L ha-1 / 2.1 L ha-1 2.1 L ha-1, 1% total tank volume |

## Slide 8
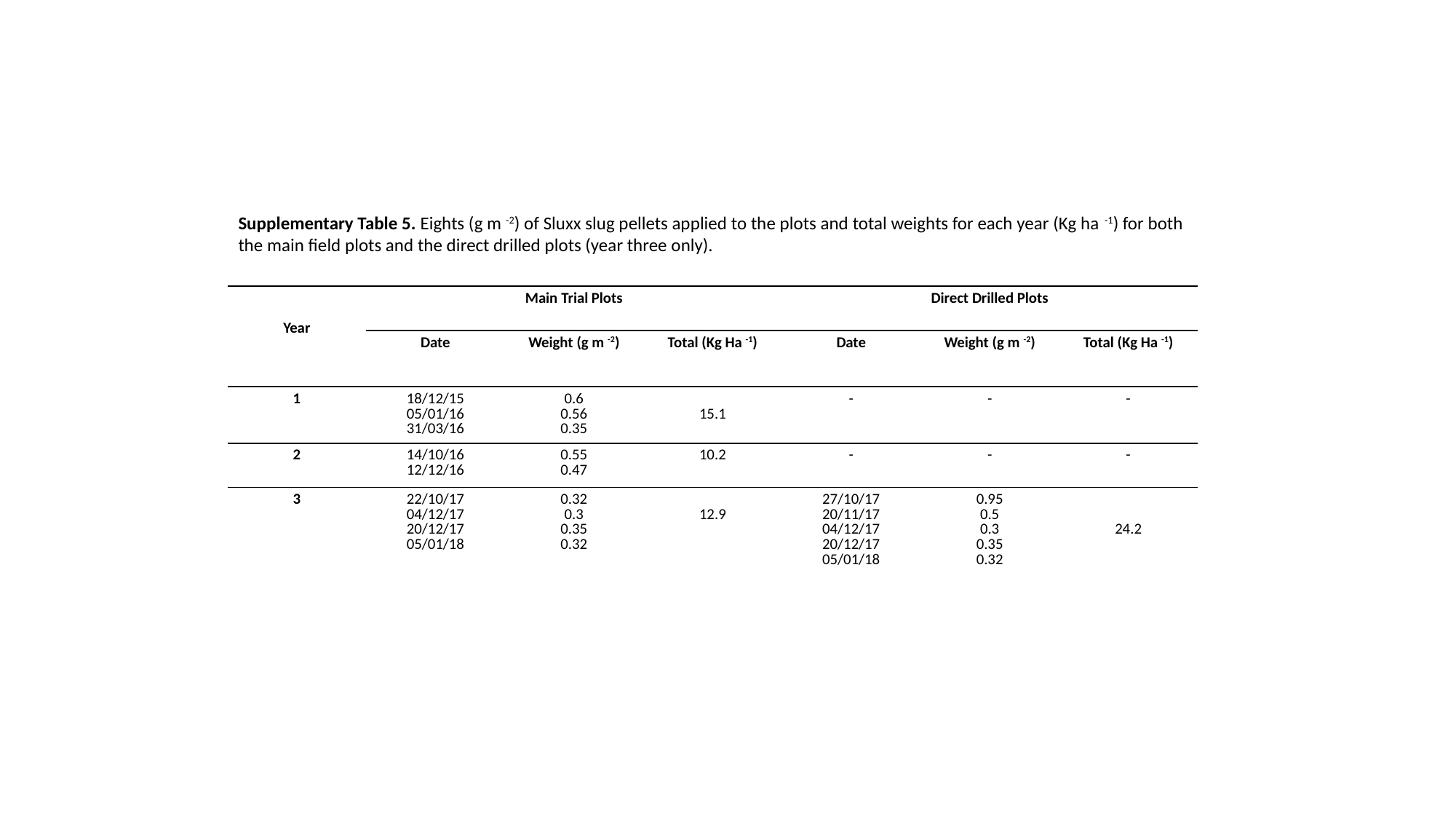

Supplementary Table 5. Eights (g m -2) of Sluxx slug pellets applied to the plots and total weights for each year (Kg ha -1) for both the main field plots and the direct drilled plots (year three only).
| Year | Main Trial Plots | | | Direct Drilled Plots | | |
| --- | --- | --- | --- | --- | --- | --- |
| | Date | Weight (g m -2) | Total (Kg Ha -1) | Date | Weight (g m -2) | Total (Kg Ha -1) |
| 1 | 18/12/15 05/01/16 31/03/16 | 0.6 0.56 0.35 | 15.1 | - | - | - |
| 2 | 14/10/16 12/12/16 | 0.55 0.47 | 10.2 | - | - | - |
| 3 | 22/10/17 04/12/17 20/12/17 05/01/18 | 0.32 0.3 0.35 0.32 | 12.9 | 27/10/17 20/11/17 04/12/17 20/12/17 05/01/18 | 0.95 0.5 0.3 0.35 0.32 | 24.2 |
